# Supplementary figures and images for: Cationic Peptides Facilitate Iron-induced Mutagenesis in Bacteria
Source: PLoS Genet. 2015 Oct 2;11(10):e1005546. doi: 10.1371/journal.pgen.1005546 (PMC4592263; doi:10.1371/journal.pgen.1005546)

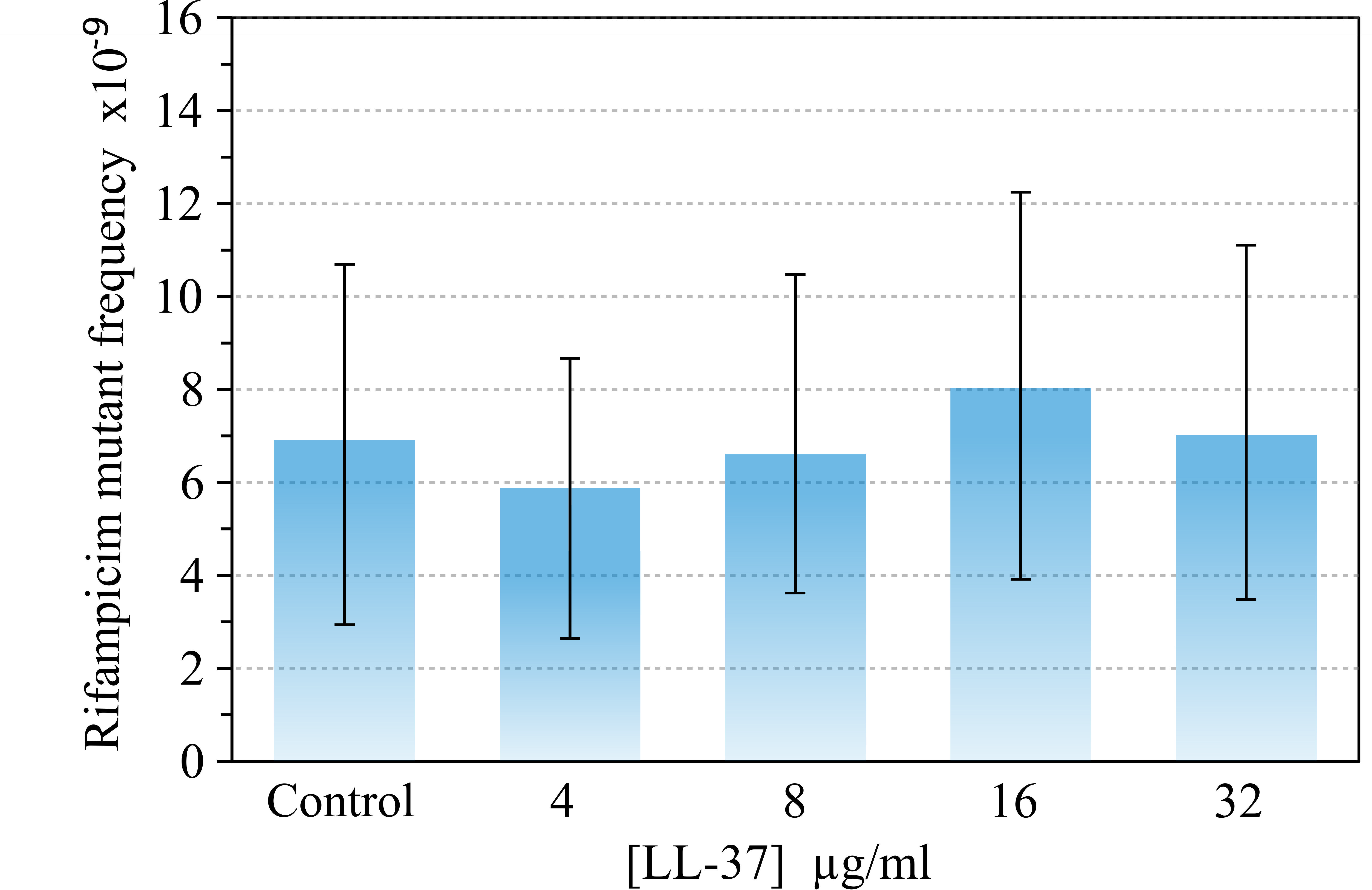

Supplement: S1 Fig — Error bars represent 95% confidence intervals for mutant frequencies. (TIFF) [file pgen.1005546.s001.tiff]

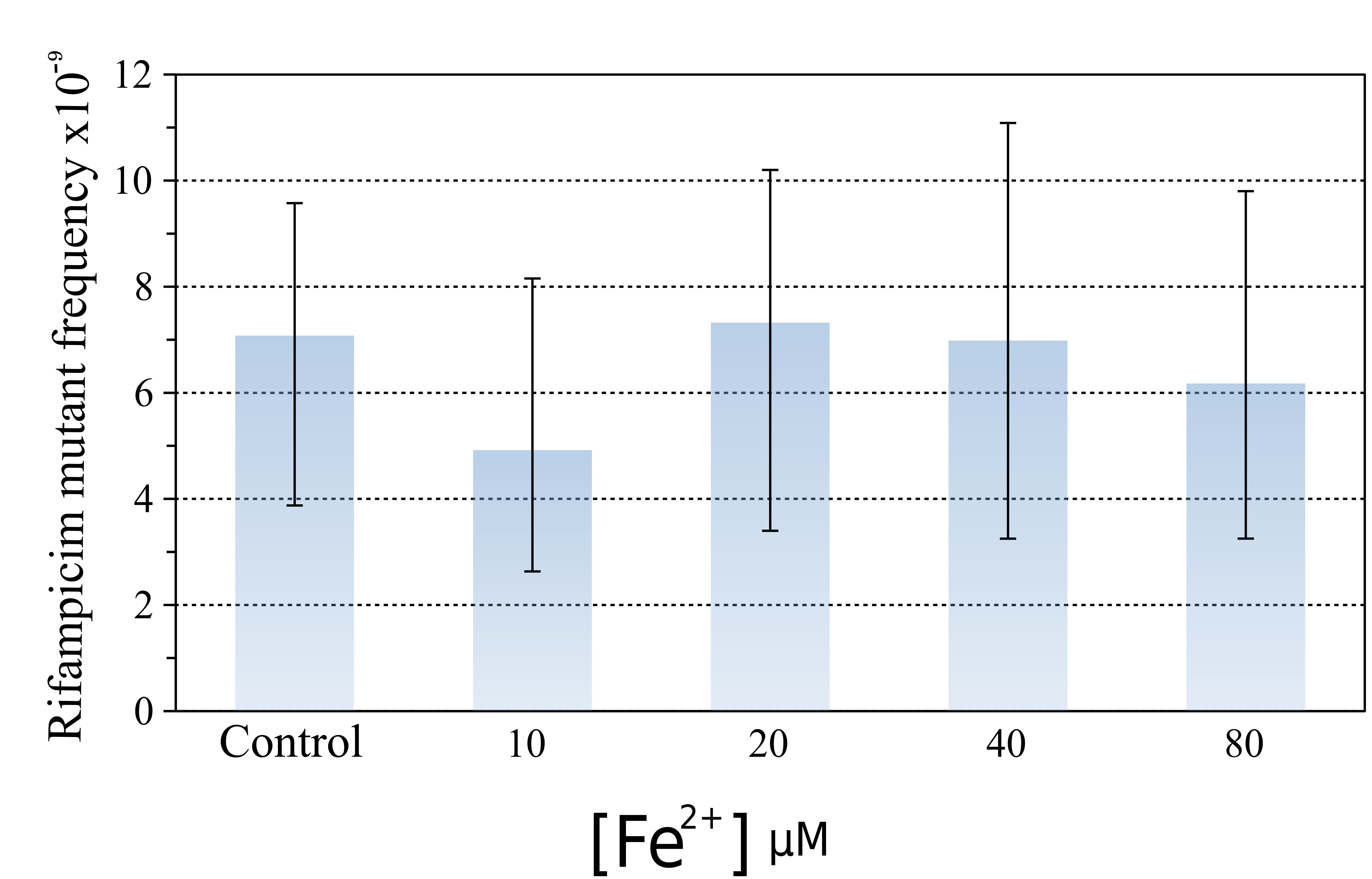

Supplement: S2 Fig — Error bars represent 95% confidence intervals for mutant frequencies. (TIFF) [file pgen.1005546.s002.tiff]

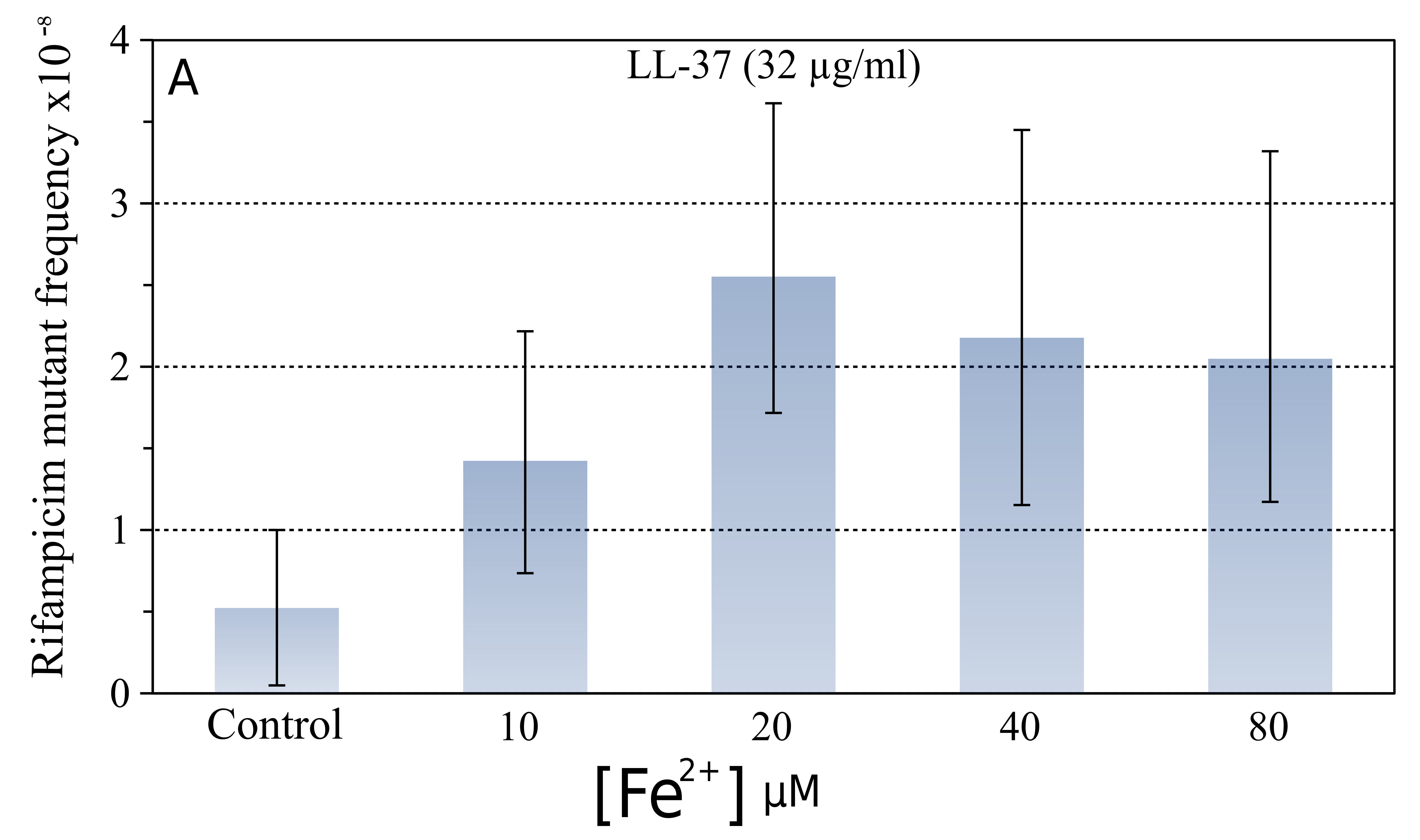

Supplement: S3 Fig — The experiment was carried out at MIC50 for LL-37 (32 μg/ml). Error bars represent 95% confidence intervals for mutant frequencies. (TIFF) [file pgen.1005546.s003.tiff]

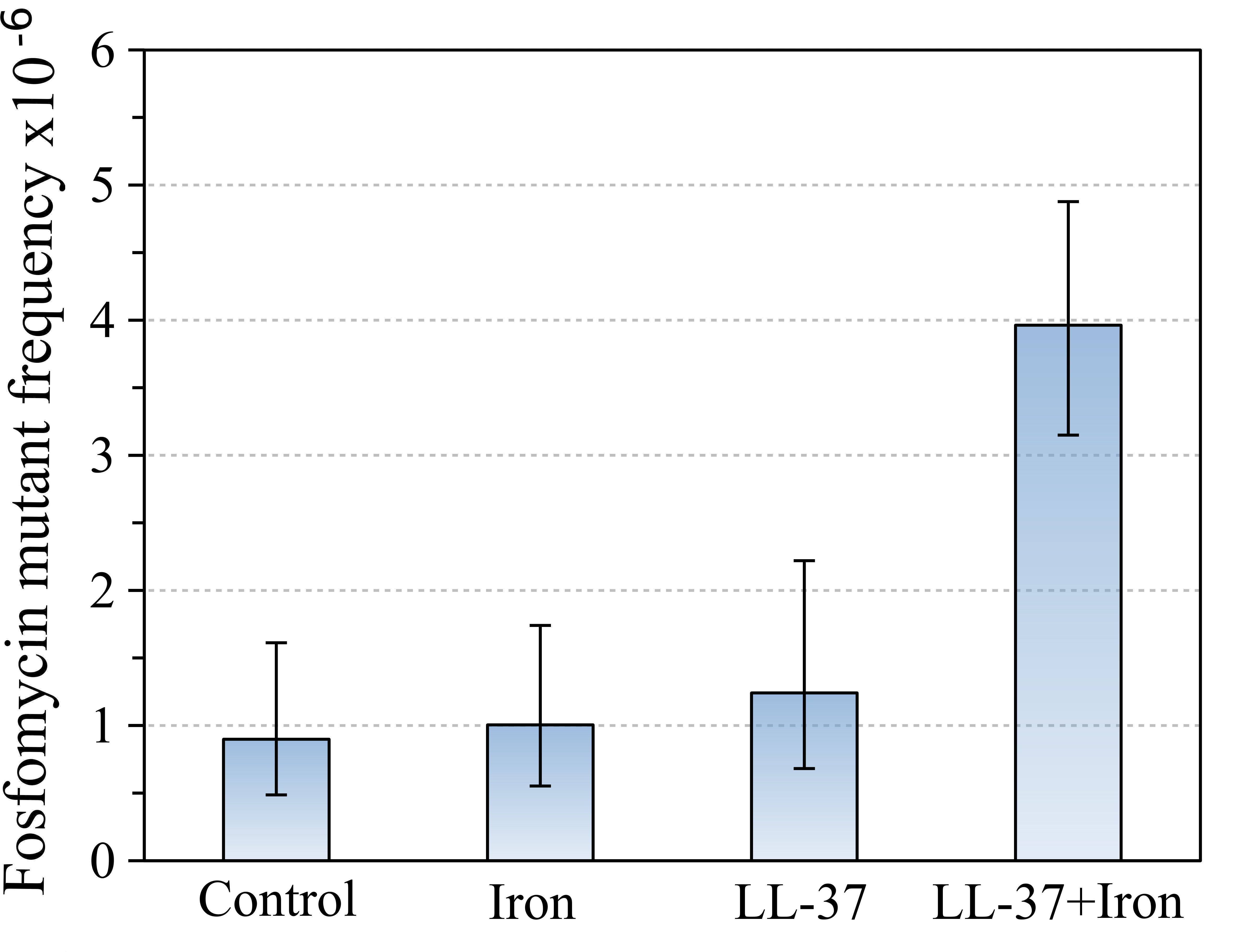

Supplement: S4 Fig — Error bars represent 95% confidence intervals for mutant frequencies. (TIFF) [file pgen.1005546.s004.tiff]

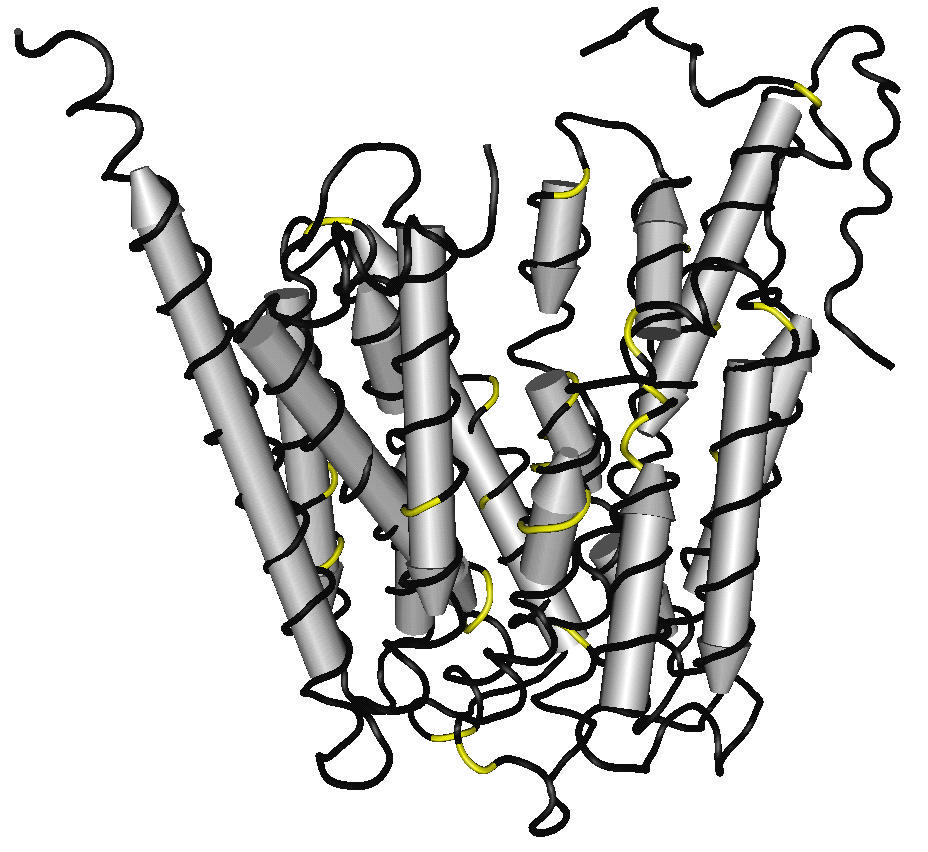

Supplement: S5 Fig — Substitutions found in Fos-R mutants in all treatments are highlighted in yellow. (TIFF) [file pgen.1005546.s005.tiff]
