# Supplementary material for: Cationic Peptides Facilitate Iron-induced Mutagenesis in Bacteria
Source: PLoS Genet. 2015 Oct 2;11(10):e1005546. doi: 10.1371/journal.pgen.1005546 (PMC4592263; doi:10.1371/journal.pgen.1005546)
Supplement: S6 Fig — The 52–1212 bp region relative to the A in the start ATG codon of the 1347bp-long glpT ORF. (PDF) [file pgen.1005546.s006.pdf]

							20							40						60							80				
WT_PA14_orf	GAG	GTG	GAC	AGC	ACC	TAC	CGG	CGC	CTG	CGC	TGG	CAG	ATC	TTC	GCC	GGT	ATC	TTC	ATC	GGC	TAC	GCG	GGT	TAC	TAC	CTG	CTG	CGC	AAG	AAC	90
iron_1	...	...	...	...	...	...	...	...	...	...	...	...	...	...	...	...	...	...	...	...	...	...	...	...	...	...	...	...	...	...	90
iron_2	...	...	...	...	...	...	...	...	...	...	...	...	...	...	...	...	...	...	...	...	...	...	...	...	...	...	...	...	...	...	90
iron_3	...	...	...	...	...	...	...	...	...	...	...	...	...	...	...	...	...	...	...	...	...	...	...	...	...	...	...	...	...	...	90
iron_4	...	...	...	...	...	...	...	...	...	...	...	...	...	...	...	...	...	...	...	...	...	...	...	...	...	...	...	...	...	...	90
iron_5	...	...	...	...	...	...	...	...	...	...	...	...	...	...	...	...	...	...	...	...	...	...	...	...	...	...	...	...	...	...	90
iron_6	...	...	...	...	...	...	...	...	...	...	...	...	...	...	...	...	...	...	...	...	...	...	...	...	...	...	...	...	...	...	90
iron_7	...	...	...	...	...	...	...	...	...	...	...	...	...	...	...	...	...	...	...	...	...	...	...	...	...	...	...	...	...	...	90
iron_8	...	...	...	...	...	...	...	...	...	...	...	...	...	...	...	...	...	...	...	...	...	...	...	...	...	...	...	...	...	...	90
iron_9	...	...	...	...	...	...	...	...	...	...	...	...	...	...	...	...	...	...	...	...	...	...	...	...	...	...	...	...	...	...	90
iron_10	...	...	...	...	...	...	...	...	...	...	...	...	...	...	...	...	...	...	...	...	...	...	...	...	...	...	...	...	...	...	90
iron_11	...	...	...	...	...	...	...	...	...	...	...	...	...	...	...	...	...	...	...	...	...	...	...	...	...	...	...	...	...	...	90
iron_12	...	...	...	...	...	...	...	...	...	...	...	...	...	...	...	...	...	...	...	...	...	...	...	...	...	...	...	...	...	...	90
iron_13	...	...	...	...	...	...	...	...	...	...	...	...	...	...	...	...	...	...	...	...	...	...	...	...	...	...	...	...	...	...	90
iron_14	...	...	...	...	...	...	...	...	...	...	...	...	...	...	...	...	...	...	...	...	...	...	...	...	...	...	...	...	...	...	90
iron_15	...	...	...	...	...	...	...	...	...	...	...	...	...	...	...	...	...	...	...	...	...	...	...	...	...	...	...	...	...	...	90
iron_16	...	...	...	...	...	...	...	...	...	...	...	...	...	...	...	...	...	...	...	...	...	...	...	...	...	...	...	...	...	...	90
iron_17	...	...	...	...	...	...	...	...	...	...	...	...	...	...	...	...	...	...	...	...	...	...	...	...	...	...	...	...	...	...	90
iron_18	...	...	...	...	...	...	...	...	...	...	...	...	...	...	...	...	...	...	...	...	...	...	...	...	...	...	...	...	...	...	90
iron_19	...	...	...	...	...	...	...	...	...	...	...	...	...	...	...	...	...	...	...	...	...	...	...	...	...	...	...	...	...	...	90
iron_20	...	...	...	...	...	...	...	...	...	...	...	...	...	...	...	...	...	...	...	...	...	...	...	...	...	...	...	...	...	...	90
LL37_1	...	...	...	...	...	...	...	...	...	...	...	...	...	...	...	...	...	...	...	...	...	...	...	...	...	...	...	...	...	...	90
LL37_2	...	...	...	...	...	...	...	...	...	...	...	...	...	...	...	...	...	...	...	...	...	...	...	...	...	...	...	...	...	...	90
LL37_3	...	...	...	...	...	...	...	...	...	...	...	...	...	...	...	...	...	...	...	...	...	...	...	...	...	...	...	...	...	...	90
LL37_4	...	...	...	...	...	...	...	...	...	...	...	...	...	...	...	...	...	...	...	...	...	...	...	...	...	...	...	...	...	...	90
LL37_5	...	...	...	...	...	...	...	...	...	...	...	...	...	...	...	...	...	...	...	...	...	...	...	...	...	...	...	...	...	...	90
LL37_6	...	...	...	...	...	...	...	...	...	...	...	...	...	...	...	...	...	...	...	...	...	...	...	...	...	...	...	...	...	...	90
LL37_7	...	...	...	...	...	...	...	...	...	...	...	...	...	...	...	...	...	...	...	...	...	...	...	...	...	...	...	...	...	...	90
LL37_8	...	...	...	...	...	...	...	...	...	...	...	...	...	...	...	...	...	...	...	...	...	...	...	...	...	...	...	...	...	...	90
LL37_9	...	...	...	...	...	...	...	...	...	...	...	...	...	...	...	...	...	...	...	...	...	...	...	...	...	...	...	...	...	...	90
LL37_10	...	...	...	...	...	...	...	...	...	...	...	...	...	...	...	.A.	...	...	...	...	...	...	...	...	...	...	...	...	...	...	90
LL37_11	...	...	...	...	...	...	...	...	...	...	...	...	...	...	...	...	...	...	...	...	...	...	...	...	...	...	...	...	...	...	90
LL37_12	...	...	...	...	...	...	...	...	...	...	...	...	...	...	...	...	...	...	...	...	...	...	...	...	...	...	...	...	...	...	90
LL37_13	...	...	...	...	...	...	...	...	...	...	...	...	...	...	...	...	...	...	...	...	...	...	...	...	...	...	...	...	...	...	90
LL37_14	...	...	...	...	...	...	...	...	...	...	...	...	...	...	...	...	...	...	...	...	...	...	...	...	...	...	...	...	...	...	90
LL37_15	...	...	...	...	...	...	...	...	...	...	...	...	...	...	...	...	...	...	...	...	...	...	...	...	...	...	...	...	...	...	90
LL37_16	...	...	...	...	...	...	...	...	...	...	...	...	...	...	...	...	...	...	...	...	...	...	...	...	...	...	...	...	...	...	90
LL37_17	...	...	...	...	...	...	...	...	...	...	...	...	...	...	...	...	...	...	...	...	...	...	...	...	...	...	...	...	...	...	90
LL37_18	...	...	...	...	...	...	...	...	...	...	...	...	...	...	...	...	...	...	...	...	...	...	...	...	...	...	...	...	...	...	90
LL37_19	...	...	...	...	...	...	...	...	...	...	...	...	...	...	...	...	...	...	...	...	...	...	...	...	...	...	...	...	...	...	90
LL37_20	...	...	...	...	...	...	...	...	...	...	...	...	...	...	...	...	...	...	...	...	...	...	...	...	...	...	...	...	...	...	90
LL37Iron_1	...	...	...	...	...	...	...	...	...	...	...	...	...	...	...	...	...	...	...	...	...	...	...	...	...	...	...	...	...	...	90
LL37Iron_2	...	...	...	...	...	...	...	...	...	...	...	...	...	...	...	...	...	...	...	...	...	...	...	...	...	...	...	...	...	...	90
LL37Iron_3	...	...	...	...	...	...	...	...	...	...	...	...	...	...	...	...	...	...	...	...	...	...	...	...	...	...	...	...	...	...	90
LL37Iron_4	...	...	...	...	...	...	...	...	...	...	...	...	...	...	...	...	...	...	...	...	...	...	...	...	...	...	...	...	...	...	90
LL37Iron_5	...	...	...	...	...	...	...	...	...	...	...	...	...	...	...	...	...	...	...	...	...	...	...	...	...	...	...	...	...	...	90
LL37Iron_6	...	...	...	...	...	...	...	...	...	...	...	...	...	...	...	...	...	...	...	...	...	...	...	...	...	...	...	...	...	...	90
LL37Iron_7	...	...	...	...	...	...	...	...	...	...	...	...	...	...	...	...	...	...	...	...	...	...	...	...	...	...	...	...	...	...	90
LL37Iron_8	...	...	...	...	...	...	...	...	...	...	...	...	...	...	...	...	...	...	...	...	...	...	...	...	...	...	...	...	...	...	90
LL37Iron_9	...	...	...	...	...	...	...	...	...	...	...	...	...	...	...	...	...	...	...	...	...	...	...	...	...	...	...	...	...	...	90
LL37Iron_10	...	...	...	...	...	...	...	...	...	...	...	...	...	...	...	...	...	...	...	...	...	...	...	...	...	...	...	...	...	...	90
LL37Iron_11	...	...	...	...	...	...	...	...	...	...	...	...	...	...	...	...	...	...	...	...	...	...	...	...	...	...	...	...	...	...	90
LL37Iron_12	...	...	...	...	...	...	...	...	...	...	...	...	...	...	...	...	...	...	...	...	...	...	...	...	...	...	...	...	...	...	90
LL37Iron_13	...	...	...	...	...	...	...	...	...	...	...	...	...	...	...	...	...	...	...	...	...	...	...	...	...	...	...	...	...	...	90
LL37Iron_14	...	...	...	...	...	...	...	...	...	...	...	...	...	...	...	...	...	...	...	...	...	...	...	...	...	...	...	...	...	...	90
LL37Iron_15	...	...	...	...	...	...	...	...	...	...	...	...	...	...	...	...	...	...	...	...	...	...	...	...	...	...	...	...	...	...	90
LL37Iron_16	...	...	...	...	...	...	...	...	...	...	...	...	...	...	...	...	...	...	...	...	...	...	...	...	...	...	...	...	...	...	90
LL37Iron_17	...	...	...	...	...	...	...	...	...	...	...	...	...	...	...	...	...	...	...	...	...	...	...	...	...	...	...	...	...	...	90

LL37Iron_18 90
LL37Iron_19 90
LL37Iron_20 90

				100						120							140							160						180	
WT_PA14_orf	TTC	TCC	CTG	GCC	ATG	CCC	TAC	CTC	ATC	GAG	GAC	GGC	TAC	AGT	CGC	GGC	CAG	CTG	GGC	GTC	GCG	ATG	TCG	GCC	ATC	GCC	ATC	GCC	TAC	GGC	180
iron_1	...	...	...	...	...	...	...	...	...	...	...	...	...	...	...	...	...	...	...	...	...	...	...	...	...	...	...	...	...	.A.	180
iron_2	...	...	...	...	...	...	...	...	...	...	...	...	...	...	...	...	...	...	...	...	...	...	...	...	...	...	---	---	...	...	174
iron_3	...	...	...	...	...	...	...	...	...	...	...	...	...	...	...	...	...	...	...	...	...	...	...	...	...	...	...	...	...	...	180
iron_4	...	...	...	...	...	...	...	...	...	...	...	...	...	...	.A.	...	...	...	...	...	...	...	...	...	...	...	...	...	...	...	180
iron_5	...	...	...	...	...	...	...	...	...	...	...	...	...	...	...	...	...	...	...	...	...	...	...	...	...	...	...	...	...	...	180
iron_6	...	...	...	...	...	...	...	...	...	...	...	...	...	...	...	...	...	...	...	...	...	...	...	...	...	...	...	...	...	...	180
iron_7	...	...	...	...	...	...	...	...	...	...	...	...	...	...	...	...	...	...	...	...	...	...	...	...	...	...	...	...	...	...	180
iron_8	...	...	...	...	...	...	...	...	...	...	...	...	...	...	...	...	...	...	...	...	...	...	...	...	...	...	...	...	...	...	180
iron_9	...	...	...	...	...	...	...	...	...	...	...	...	...	...	...	...	...	...	...	...	...	...	...	...	...	...	...	...	...	...	180
iron_10	...	...	...	...	...	...	...	...	...	...	...	...	...	...	...	...	...	...	...	...	...	...	...	...	...	...	---	---	...	...	174
iron_11	...	...	...	...	...	...	...	...	...	...	...	...	...	...	...	...	...	...	...	...	...	...	...	...	...	...	---	---	...	...	174
iron_12	...	...	.C.	...	...	...	...	...	...	...	...	...	...	...	...	...	...	...	...	...	...	...	...	...	...	...	...	...	...	...	180
iron_13	...	...	...	...	...	...	...	...	...	...	...	...	...	...	...	...	...	...	...	...	...	...	...	...	...	...	---	---	...	...	174
iron_14	...	...	...	...	...	...	...	...	...	...	...	...	...	...	...	...	...	...	...	...	...	...	...	...	...	...	---	---	...	...	174
iron_15	...	...	...	...	...	...	...	...	...	...	...	...	...	...	...	...	...	...	...	...	...	...	...	...	...	...	---	---	...	...	174
iron_16	...	...	...	...	...	...	...	...	...	...	...	...	...	...	...	...	...	...	...	...	...	...	...	...	...	...	---	---	...	...	174
iron_17	...	...	...	...	...	...	...	...	...	...	...	...	...	...	...	...	...	...	...	...	...	...	...	...	...	...	---	---	...	...	174
iron_18	...	...	...	...	...	...	...	...	...	...	...	...	...	...	...	...	...	...	...	...	...	...	...	...	...	...	...	...	...	...	180
iron_19	...	...	...	...	...	...	...	...	...	...	...	...	...	...	...	...	...	...	...	...	...	...	...	...	...	...	...	...	...	...	180
iron_20	...	...	...	...	...	...	...	...	...	...	...	...	...	...	...	...	...	...	...	...	...	...	...	...	...	...	...	...	...	...	180
LL37_1	...	...	...	...	...	...	...	...	...	...	...	...	...	...	...	...	...	...	...	...	...	...	...	...	...	...	...	...	...	...	180
LL37_2	...	...	...	...	...	...	...	...	...	...	...	...	...	...	...	...	...	...	...	...	...	...	...	...	...	...	---	---	...	...	174
LL37_3	...	...	...	...	...	...	...	...	...	...	...	...	...	...	...	...	...	...	...	...	...	...	...	...	...	...	...	...	...	...	180
LL37_4	...	...	...	...	...	...	...	...	...	...	...	...	...	...	...	...	...	...	...	...	...	...	...	...	...	...	...	...	...	...	180
LL37_5	...	...	...	...	...	...	...	...	...	...	...	...	...	...	...	...	...	...	...	...	...	...	...	...	...	...	---	---	...	...	174
LL37_6	...	...	...	...	...	...	...	...	...	...	...	...	...	...	...	...	...	...	...	...	...	...	...	...	...	...	...	...	...	...	180
LL37_7	...	...	...	...	...	...	...	...	...	...	...	...	...	...	...	...	...	...	...	...	...	...	...	...	...	...	---	---	...	...	174
LL37_8	...	...	...	...	...	...	...	...	...	...	...	...	...	...	...	...	...	...	...	...	...	...	...	...	...	...	...	...	...	...	180
LL37_9	...	...	...	...	...	...	...	...	...	...	...	...	...	...	...	...	...	...	...	...	...	...	...	...	...	...	---	---	...	...	174
LL37_10	...	...	...	...	...	...	...	...	...	...	...	...	...	...	...	...	...	...	...	...	...	...	...	...	...	...	...	...	...	...	180
LL37_11	...	...	...	...	...	...	...	...	...	...	...	...	...	...	...	...	...	...	...	...	...	...	...	...	...	...	---	---	...	...	174
LL37_12	...	...	...	...	...	...	...	...	...	...	...	...	...	...	...	...	...	...	...	...	...	...	...	...	...	...	...	...	...	...	180
LL37_13	...	...	...	...	...	...	...	...	...	...	...	...	...	...	...	...	...	...	...	...	...	...	...	...	...	...	...	...	...	...	180
LL37_14	...	...	...	...	...	...	...	...	...	...	...	...	...	...	...	...	...	...	...	...	...	...	...	...	...	...	...	...	...	...	180
LL37_15	...	...	...	...	...	...	...	...	...	...	...	...	...	...	...	...	...	...	...	...	...	...	...	...	...	...	...	...	...	...	180
LL37_16	...	...	...	...	...	...	...	...	...	...	...	...	...	...	...	...	...	...	...	...	...	...	...	...	...	...	...	...	...	...	180
LL37_17	...	...	...	...	...	...	...	...	...	...	...	...	...	...	...	...	...	...	...	...	...	...	...	...	...	...	...	...	...	...	180
LL37_18	...	...	...	...	...	...	...	...	...	...	...	...	...	...	...	...	...	...	...	...	...	...	...	...	...	...	---	---	...	...	174
LL37_19	...	...	...	...	...	...	...	...	...	...	...	...	...	...	...	...	...	...	...	...	...	...	...	...	...	...	...	...	...	...	180
LL37_20	...	...	...	...	...	...	...	...	...	...	...	...	...	...	...	...	...	...	...	...	...	...	...	...	...	...	...	...	...	A..	180
LL37Iron_1	...	...	...	...	...	...	...	...	...	...	...	...	...	...	...	...	...	...	...	...	...	...	...	...	...	...	...	...	...	...	180
LL37Iron_2	...	...	...	...	...	...	...	...	...	...	...	...	...	...	...	...	...	...	...	...	...	...	...	...	...	...	...	...	...	...	180
LL37Iron_3	...	...	...	...	...	...	...	...	...	...	...	...	...	...	...	...	...	...	...	...	...	...	...	...	...	...	...	...	...	...	180
LL37Iron_4	...	...	...	...	...	...	...	...	...	...	...	...	...	...	...	...	...	...	...	...	...	...	...	...	...	...	...	...	...	...	180
LL37Iron_5	...	...	...	...	...	...	...	...	...	...	...	...	...	...	...	...	...	...	...	...	...	...	...	...	...	...	...	...	...	...	180
LL37Iron_6	...	...	...	...	...	...	...	...	...	...	...	...	...	...	...	...	...	...	...	...	...	...	...	...	...	...	...	...	...	...	180
LL37Iron_7	...	...	...	...	...	...	...	...	...	...	...	...	...	...	...	...	...	...	...	...	...	...	...	...	...	...	...	...	...	...	180
LL37Iron_8	...	...	...	...	...	...	...	...	...	...	...	...	...	...	...	...	...	...	...	...	...	...	...	...	...	...	...	...	...	...	180
LL37Iron_9	...	...	...	...	...	...	...	...	...	...	...	...	...	...	...	...	...	...	...	...	...	...	...	...	...	...	...	...	...	...	180
LL37Iron_10	...	...	...	...	...	...	...	...	...	...	...	...	...	...	...	...	...	...	...	...	...	...	...	...	...	...	...	...	...	...	180
LL37Iron_11	...	...	...	...	...	...	...	...	...	...	...	...	...	...	...	...	...	...	...	...	...	...	...	...	...	...	...	...	...	...	180
LL37Iron_12	...	...	...	...	...	...	...	...	...	...	...	...	...	...	...	...	...	...	...	...	...	...	...	...	...	...	...	...	...	...	180
LL37Iron_13	...	...	...	...	...	...	...	...	...	...	...	...	...	...	...	...	...	...	...	...	...	...	...	...	...	...	...	...	...	...	180
LL37Iron_14	...	...	...	...	...	...	...	...	...	...	...	...	...	...	...	...	...	...	...	...	...	...	...	...	...	...	...	...	...	...	180
LL37Iron_15	...	...	...	...	...	...	...	...	...	...	...	...	...	...	...	...	...	...	...	...	...	...	...	...	...	...	...	...	...	...	180
LL37Iron_16	...	...	...	...	...	...	...	...	...	...	...	...	...	...	...	...	...	...	...	...	...	...	...	...	...	...	...	...	...	...	180
LL37Iron_17	...	...	...	...	...	...	...	...	...	...	...	...	...	...	...	...	...	...	...	...	...	...	...	...	...	...	...	...	...	...	180

LL37Iron_18 180
LL37Iron_19 180
LL37Iron_20 180

							200							220						240							260				
WT_PA14_orf	CTG	TCG	AAA	TTC	ATC	ATG	GGC	CTG	GTC	TCG	GAC	CGT	TCC	AAC	CCA	CGG	GTC	TTC	CTG	CCG	TTC	GGC	CTG	TTG	GTG	TCG	GCG	GCG	GTG	ATG	270
iron_1	...	...	...	...	...	...	...	...	...	...	...	...	...	...	...	...	...	...	...	...	...	...	...	...	...	...	...	...	...	...	270
iron_2	...	...	...	...	...	...	...	...	...	...	...	...	...	...	...	...	...	...	...	...	...	...	...	...	...	...	...	...	...	...	264
iron_3	...	...	...	...	...	...	...	...	...	...	...	...	...	...	...	...	...	...	...	...	...	...	...	...	...	...	...	...	...	...	270
iron_4	...	...	...	...	...	...	...	...	...	...	...	...	...	...	...	...	...	...	...	...	...	...	...	...	...	...	...	...	...	...	270
iron_5	...	...	...	...	...	...	...	...	...	...	...	...	...	...	...	...	...	...	...	...	...	...	...	...	...	...	...	...	...	...	270
iron_6	...	...	...	...	...	...	T..	...	...	...	...	...	...	...	...	...	...	...	...	...	...	...	...	...	...	...	...	...	...	...	270
iron_7	...	...	...	...	...	...	...	...	...	...	...	...	...	...	...	...	...	...	...	...	...	...	...	...	...	...	...	...	...	...	270
iron_8	...	...	...	...	...	...	...	...	...	...	...	...	...	...	...	...	...	...	...	...	...	...	...	...	...	...	...	...	...	...	270
iron_9	...	...	...	...	...	...	...	...	...	...	...	...	...	...	...	...	...	...	...	...	...	...	...	...	...	...	...	...	...	...	270
iron_10	...	...	...	...	...	...	...	...	...	...	...	...	...	...	...	...	...	...	...	...	...	...	...	...	...	...	...	...	...	...	264
iron_11	...	...	...	...	...	...	...	...	...	...	...	...	...	...	...	...	...	...	...	...	...	...	...	...	...	...	...	...	...	...	264
iron_12	...	...	...	...	...	...	...	...	...	...	...	...	...	...	...	...	...	...	...	...	...	...	...	...	...	...	...	...	...	...	270
iron_13	...	...	...	...	...	...	...	...	...	...	...	...	...	...	...	...	...	...	...	...	...	...	...	...	...	...	...	...	...	...	264
iron_14	...	...	...	...	...	...	...	...	...	...	...	...	...	...	...	...	...	...	...	...	...	...	...	...	...	...	...	...	...	...	264
iron_15	...	...	...	...	...	...	...	...	...	...	...	...	...	...	...	...	...	...	...	...	...	...	...	...	...	...	...	...	...	...	264
iron_16	...	...	...	...	...	...	...	...	...	...	...	...	...	...	...	...	...	...	...	...	...	...	...	...	...	...	...	...	...	...	264
iron_17	...	...	...	...	...	...	...	...	...	...	...	...	...	...	...	...	...	...	...	...	...	...	...	...	...	...	...	...	...	...	264
iron_18	...	...	...	...	...	...	...	...	...	...	...	...	...	...	...	...	...	...	...	...	...	...	...	...	...	...	...	...	...	...	270
iron_19	...	...	...	...	...	...	...	...	...	...	...	...	...	...	...	...	...	...	...	...	...	...	...	...	...	...	...	...	...	...	270
iron_20	...	...	...	...	...	...	...	...	...	...	...	...	...	...	...	...	...	...	...	...	...	...	...	...	...	...	...	...	...	...	270
LL37_1	...	...	...	...	...	...	...	...	...	...	...	...	...	...	...	...	...	...	...	...	...	...	...	...	...	...	...	...	...	...	270
LL37_2	...	...	...	...	...	...	...	...	...	...	...	...	...	...	...	...	...	...	...	...	...	...	...	...	...	...	...	...	...	...	264
LL37_3	...	...	...	...	...	...	...	...	...	...	...	...	...	...	...	...	...	...	...	...	...	...	...	...	...	...	...	...	...	...	270
LL37_4	...	...	...	...	...	...	...	...	...	...	...	...	...	...	...	...	...	...	...	...	...	...	...	...	...	...	...	...	...	...	270
LL37_5	...	...	...	...	...	...	...	...	...	...	...	...	...	...	...	...	...	...	...	...	...	...	...	...	...	...	...	...	...	...	264
LL37_6	...	...	...	...	...	...	...	...	...	...	...	...	...	...	...	...	...	...	...	...	...	...	...	...	...	...	...	...	...	...	270
LL37_7	...	...	...	...	...	...	...	...	...	...	...	...	...	...	...	...	...	...	...	...	...	...	...	...	...	...	...	...	...	...	264
LL37_8	...	...	...	...	...	...	...	...	...	...	...	...	...	...	...	...	...	...	...	...	...	...	...	...	...	...	...	...	...	...	270
LL37_9	...	...	...	...	...	...	...	...	...	...	...	...	...	...	...	...	...	...	...	...	...	...	...	...	...	...	...	...	...	...	264
LL37_10	...	...	...	...	...	...	...	...	...	...	...	...	...	...	...	...	...	...	...	...	...	...	...	...	...	...	...	...	...	...	270
LL37_11	...	...	...	...	...	...	...	...	...	...	...	...	...	...	...	...	...	...	...	...	...	...	...	...	...	...	...	...	...	...	264
LL37_12	...	...	...	...	...	...	...	...	...	...	...	...	...	...	...	...	...	...	...	...	...	...	...	...	...	...	...	...	...	...	270
LL37_13	...	...	...	...	...	...	...	...	...	...	...	...	...	...	...	...	...	...	...	...	...	...	...	...	...	...	...	...	...	...	270
LL37_14	...	...	...	...	...	...	...	...	...	...	...	...	...	...	...	...	...	...	...	...	...	...	...	...	...	...	...	...	...	...	270
LL37_15	...	...	...	...	...	...	...	...	...	...	...	...	...	...	...	...	...	...	...	...	...	...	...	...	...	...	...	...	...	...	270
LL37_16	...	...	...	...	...	...	...	...	...	...	...	...	...	...	...	...	...	...	...	...	...	...	...	...	...	...	...	...	...	...	270
LL37_17	...	...	...	...	...	...	...	...	...	...	...	...	...	...	...	...	...	...	...	...	...	...	...	...	...	...	...	...	...	...	270
LL37_18	...	...	...	...	...	...	...	...	...	...	...	...	...	...	...	...	...	...	...	...	...	...	...	...	...	...	...	...	...	...	264
LL37_19	...	...	...	...	...	...	...	...	...	...	...	...	...	...	...	...	...	...	...	...	...	...	...	...	...	...	...	...	...	...	270
LL37_20	...	...	...	...	...	...	...	...	...	...	...	...	...	...	...	...	...	...	...	...	...	...	...	...	...	...	...	...	...	...	270
LL37Iron_1	...	...	...	...	...	...	...	...	...	...	...	...	...	...	...	...	...	...	...	...	...	...	...	...	...	...	...	...	...	...	270
LL37Iron_2	...	...	...	...	...	...	...	...	...	...	...	...	...	...	...	T..	...	...	...	...	...	...	...	...	...	...	...	...	...	...	270
LL37Iron_3	...	...	...	...	...	...	...	...	...	...	...	...	...	...	...	...	...	...	...	...	...	...	...	...	...	...	...	...	...	...	270
LL37Iron_4	...	...	...	...	...	...	...	...	...	...	...	...	...	...	...	T..	...	...	...	...	...	...	...	...	...	...	...	...	...	...	270
LL37Iron_5	...	...	...	...	...	...	...	...	...	...	...	...	...	...	...	T..	...	...	...	...	...	...	...	...	...	...	...	...	...	...	270
LL37Iron_6	...	...	...	...	...	...	...	...	...	...	...	...	...	...	...	...	...	...	...	...	...	...	...	...	...	...	...	...	...	...	270
LL37Iron_7	...	...	...	...	...	...	...	...	...	...	...	...	...	...	...	T..	...	...	...	...	...	...	...	...	...	...	...	...	...	...	270
LL37Iron_8	...	...	...	...	...	...	...	...	...	...	...	...	...	...	...	T..	...	...	...	...	...	...	...	...	...	...	...	...	...	...	270
LL37Iron_9	...	...	...	...	...	...	...	...	...	...	...	...	...	...	...	T..	...	...	...	...	...	...	...	...	...	...	...	...	...	...	270
LL37Iron_10	...	...	...	...	...	...	...	...	...	...	...	...	...	...	...	...	...	...	...	...	...	...	...	...	...	...	...	...	...	...	270
LL37Iron_11	...	...	...	...	...	...	...	...	...	...	...	...	...	...	...	...	...	...	...	...	...	...	...	...	...	...	...	...	...	...	270
LL37Iron_12	...	...	...	...	...	...	...	...	...	...	...	...	...	...	...	...	...	...	...	...	...	...	...	...	...	...	...	...	...	...	270
LL37Iron_13	...	...	...	...	...	...	...	...	...	...	...	...	...	...	...	...	...	...	...	...	...	...	...	...	...	...	...	...	...	...	270
LL37Iron_14	...	...	...	...	...	...	...	...	...	...	...	...	...	...	...	T..	...	...	...	...	...	...	...	...	...	...	...	...	...	...	270
LL37Iron_15	...	...	...	...	...	...	...	...	...	...	...	...	...	...	...	T..	...	...	...	...	...	...	...	...	...	...	...	...	...	...	270
LL37Iron_16	...	...	...	...	...	...	...	...	...	...	...	...	...	...	...	T..	...	...	...	...	...	...	...	...	...	...	...	...	...	...	270
LL37Iron_17	...	...	...	...	...	...	...	...	...	...	...	...	...	...	...	T..	...	...	...	...	...	...	...	...	...	...	...	...	...	...	270

```
LL37Iron_18  ... ... ... ... ... ... ... ... ... ... ... ... ... ... ... ... ... ... ... ... ... ... .-. ... ... ... ... ... ... .... 269
LL37Iron_19  ... ... ... ... ... ... ... ... ... ... ... ... ... ... ... ... ... ... ... ... ... ... ... ... ... ... ... ... ... .... 270
LL37Iron_20  ... ... ... ... ... ... ... ... ... ... ... ... ... ... ... ... ... ... ... ... ... ... ... ... ... ... ... ... ... .... 270
```

				280						300							320							340						360	
WT_PA14_orf	TTC	ATT	TTC	GGA	TTC	GCG	CAC	TGG	GCT	ACC	TCC	AGC	GTC	GCC	ATC	ATG	TTC	GTC	CTG	CTC	TTC	ATT	AAC	GGC	TGG	GCC	CAG	GGC	ATG	GGC	360
iron_1	. . .	. . .	. . .	. . .	. . .	. . .	. . .	. . .	. . .	. . .	. . .	. . .	. . .	. . .	. . .	. . .	. . .	. . .	. . .	. . .	. . .	. . .	. . .	. . .	. . .	. . .	. . .	. . .	. . .	. . .	360
iron_2	. . .	. . .	. . .	. . .	. . .	. . .	. . .	. . .	. . .	. . .	. . .	. . .	. . .	. . .	. . .	. . .	. . .	. . .	. . .	. . .	. . .	. . .	. . .	. . .	. . .	. . .	. . .	. . .	. . .	. . .	354
iron_3	. . .	. . .	. . .	. . .	. . .	. . .	. . .	. . .	. . .	. . .	. . .	. . .	. . .	. . .	. . .	. . .	. . .	. . .	. . .	. . .	. . .	. . .	. . .	. . .	. . .	. . .	. . .	. . .	. . .	. . .	360
iron_4	. . .	. . .	. . .	. . .	. . .	. . .	. . .	. . .	. . .	. . .	. . .	. . .	. . .	. . .	. . .	. . .	. . .	. . .	. . .	. . .	. . .	. . .	. . .	. . .	. . .	. . .	. . .	. . .	. . .	. . .	360
iron_5	. . .	. . .	. . .	. . .	. . .	. . .	. . .	. . .	. . .	. . .	. . .	. . .	. . .	. . .	. . .	. . .	. . .	. . .	. . .	. . .	. . .	. . .	. . .	. . .	. . .	. . .	. . .	. . .	. . .	. . .	360
iron_6	. . .	. . .	. . .	. . .	. . .	. . .	. . .	. . .	. . .	. . .	. . .	. . .	. . .	. . .	. . .	. . .	. . .	. . .	. . .	. . .	. . .	. . .	. . .	. . .	. . .	. . .	. . .	. . .	. . .	. . .	360
iron_7	. . .	. . .	. . .	. . .	. . .	. . .	. . .	. . .	. . .	. . .	. . .	. . .	. . .	. . .	. . .	. . .	. . .	. . .	. . .	. . .	. . .	. . .	. . .	. . .	. . .	. . .	. . .	. . .	. . .	A . .	360
iron_8	. . .	. . .	. . .	. . .	. . .	. . .	. . .	. . .	. . .	. . .	. . .	. . .	. . .	. . .	. . .	. . .	. . .	. . .	. . .	. . .	. . .	. . .	. . .	. . .	. . .	. . .	. . .	. . .	. . .	. . .	360
iron_9	. . .	. . .	. . .	. . .	. . .	. . .	. . .	. . .	. . .	. . .	. . .	. . .	. . .	. . .	. . .	. . .	. . .	. . .	. . .	. . .	. . .	. . .	. . .	. . .	. . .	. . .	. . .	. . .	. . .	. . .	360
iron_10	. . .	. . .	. . .	. . .	. . .	. . .	. . .	. . .	. . .	. . .	. . .	. . .	. . .	. . .	. . .	. . .	. . .	. . .	. . .	. . .	. . .	. . .	. . .	. . .	. . .	. . .	. . .	. . .	. . .	. . .	354
iron_11	. . .	. . .	. . .	. . .	. . .	. . .	. . .	. . .	. . .	. . .	. . .	. . .	. . .	. . .	. . .	. . .	. . .	. . .	. . .	. . .	. . .	. . .	. . .	. . .	. . .	. . .	. . .	. . .	. . .	. . .	354
iron_12	. . .	. . .	. . .	. . .	. . .	. . .	. . .	. . .	. . .	. . .	. . .	. . .	. . .	. . .	. . .	. . .	. . .	. . .	. . .	. . .	. . .	. . .	. . .	. . .	. . .	. . .	. . .	. . .	. . .	. . .	360
iron_13	. . .	. . .	. . .	. . .	. . .	. . .	. . .	. . .	. . .	. . .	. . .	. . .	. . .	. . .	. . .	. . .	. . .	. . .	. . .	. . .	. . .	. . .	. . .	. . .	. . .	. . .	. . .	. . .	. . .	. . .	354
iron_14	. . .	. . .	. . .	. . .	. . .	. . .	. . .	. . .	. . .	. . .	. . .	. . .	. . .	. . .	. . .	. . .	. . .	. . .	. . .	. . .	. . .	. . .	. . .	. . .	. . .	. . .	. . .	. . .	. . .	. . .	354
iron_15	. . .	. . .	. . .	. . .	. . .	. . .	. . .	. . .	. . .	. . .	. . .	. . .	. . .	. . .	. . .	. . .	. . .	. . .	. . .	. . .	. . .	. . .	. . .	. . .	. . .	. . .	. . .	. . .	. . .	. . .	354
iron_16	. . .	. . .	. . .	. . .	. . .	. . .	. . .	. . .	. . .	. . .	. . .	. . .	. . .	. . .	. . .	. . .	. . .	. . .	. . .	. . .	. . .	. . .	. . .	. . .	. . .	. . .	. . .	. . .	. . .	. . .	354
iron_17	. . .	. . .	. . .	. . .	. . .	. . .	. . .	. . .	. . .	. . .	. . .	. . .	. . .	. . .	. . .	. . .	. . .	. . .	. . .	. . .	. . .	. . .	. . .	. . .	. . .	. . .	. . .	. . .	. . .	. . .	354
iron_18	. . .	. . .	. . .	. . .	. . .	. . .	. . .	. . .	. . .	. . .	. . .	. . .	. . .	. . .	. . .	. . .	. . .	. . .	. . .	. . .	. . .	. . .	. . .	. . .	. . .	. . .	. . .	. . .	. . .	T . .	360
iron_19	. . .	. . .	. . .	. . .	. . .	. . .	. . .	. . .	. . .	. . .	. . .	. . .	. . .	. . .	. . .	. . .	. . .	. . .	. . .	. . .	. . .	. . .	. . .	. . .	. . .	. . .	. . .	. . .	. . .	. . .	360
iron_20	. . .	. . .	. . .	. . .	. . .	. . .	. . .	. . .	. . .	. . .	. . .	. . .	. . .	. . .	. . .	. . .	. . .	. . .	. . .	. . .	. . .	. . .	. . .	. . .	. . .	. . .	. . .	. . .	. . .	. . .	360
LL37_1	. . .	. . .	. . .	. . .	. . .	. . .	. . .	. . .	. . .	. . .	. . .	. . .	. . .	. . .	. . .	. . .	. . .	. . .	. . .	. . .	. . .	. . .	. . .	. . .	. . .	. . .	. . .	. . .	. . .	. . .	360
LL37_2	. . .	. . .	. . .	. . .	. . .	. . .	. . .	. . .	. . .	. . .	. . .	. . .	. . .	. . .	. . .	. . .	. . .	. . .	. . .	. . .	. . .	. . .	. . .	. . .	. . .	. . .	. . .	. . .	. . .	. . .	354
LL37_3	. . .	. . .	. . .	. . .	. . .	. . .	. . .	. . .	. . .	. . .	. . .	. . .	. . .	. . .	. . .	. . .	. . .	. . .	. . .	. . .	. . .	. . .	. . .	. . .	. . .	. . .	. . .	. . .	. . .	. . .	360
LL37_4	. . .	. . .	. . .	. . .	. . .	. . .	. . .	. . .	. . .	. . .	. . .	. . .	. . .	. . .	. . .	. . .	. . .	. . .	. . .	. . .	. . .	. . .	. . .	. . .	. . .	. . .	. T .	. . .	. . .	. . .	360
LL37_5	. . .	. . .	. . .	. . .	. . .	. . .	. . .	. . .	. . .	. . .	. . .	. . .	. . .	. . .	. . .	. . .	. . .	. . .	. . .	. . .	. . .	. . .	. . .	. . .	. . .	. . .	. . .	. . .	. . .	. . .	354
LL37_6	. . .	. . .	. . .	. . .	. . .	. . .	. . .	. . .	. . .	. . .	. . .	. . .	. . .	. . .	. . .	. . .	. . .	. . .	. . .	. . .	. . .	. . .	. . .	. . .	. . .	. . .	. . .	. . .	. . .	. . .	360
LL37_7	. . .	. . .	. . .	. . .	. . .	. . .	. . .	. . .	. . .	. . .	. . .	. . .	. . .	. . .	. . .	. . .	. . .	. . .	. . .	. . .	. . .	. . .	. . .	. . .	. . .	. . .	. . .	. . .	. . .	. . .	354
LL37_8	. . .	. . .	. . .	. . .	. . .	. . .	. . .	. . .	. . .	. . .	. . .	. . .	. . .	. . .	. . .	. . .	. . .	. . .	. . .	. . .	. . .	. . .	. . .	. . .	. . .	. . .	. . .	. . .	. . .	. . .	360
LL37_9	. . .	. . .	. . .	. . .	. . .	. . .	. . .	. . .	. . .	. . .	. . .	. . .	. . .	. . .	. . .	. . .	. . .	. . .	. . .	. . .	. . .	. . .	. . .	. . .	. . .	. . .	. . .	. . .	. . .	. . .	354
LL37_10	. . .	. . .	. . .	. . .	. . .	. . .	. . .	. . .	. . .	. . .	. . .	. . .	. . .	. . .	. . .	. . .	. . .	. . .	. . .	. . .	. . .	. . .	. . .	. . .	. . .	. . .	. . .	. . .	. . .	. . .	360
LL37_11	. . .	. . .	. . .	. . .	. . .	. . .	. . .	. . .	. . .	. . .	. . .	. . .	. . .	. . .	. . .	. . .	. . .	. . .	. . .	. . .	. . .	. . .	. . .	. . .	. . .	. . .	. . .	. . .	. . .	. . .	354
LL37_12	. . .	. . .	. . .	. . .	. . .	. . .	. . .	. . .	. . .	. . .	. . .	. . .	. . .	. . .	. . .	. . .	. . .	. . .	. . .	. . .	. . .	. . .	. . .	. . .	. . .	. . .	. . .	. . .	. . .	. . .	360
LL37_13	. . .	. . .	. . .	. . .	. . .	. . .	. . .	. . .	. . .	. . .	. . .	. . .	. . .	. . .	. . .	. . .	. . .	. . .	. . .	. . .	. . .	. . .	. . .	. . .	. . .	. . .	. . .	. . .	. . .	. . .	360
LL37_14	. . .	. . .	. . .	. . .	. . .	. . .	. . .	. . .	. . .	. . .	. . .	. . .	. . .	. . .	. . .	. . .	. . .	. . .	. . .	. . .	. . .	. . .	. . .	. . .	. . .	. . .	. . .	. . .	. . .	T . .	360
LL37_15	. . .	. . .	. . .	. . .	. . .	. . .	. . .	. . .	. . .	. . .	. . .	. . .	. . .	. . .	. . .	. . .	. . .	. . .	. . .	. . .	. . .	. . .	. . .	. . .	. . .	. . .	. . .	. . .	. . .	. . .	360
LL37_16	. . .	. . .	. . .	. . .	. . .	. . .	. . .	. . .	. . .	. . .	. . .	. . .	. . .	. . .	. . .	. . .	. . .	. . .	. . .	. . .	. . .	. . .	. . .	. . .	. . .	. . .	. . .	. . .	. . .	. . .	360
LL37_17	. . .	. . .	. . .	. . .	. . .	. . .	. . .	. . .	. . .	. . .	. . .	. . .	. . .	. . .	. . .	. . .	. . .	. . .	. . .	. . .	. . .	. . .	. . .	. . .	. . .	. . .	. . .	. . .	. . .	. . .	360
LL37_18	. . .	. . .	. . .	. . .	. . .	. . .	. . .	. . .	. . .	. . .	. . .	. . .	. . .	. . .	. . .	. . .	. . .	. . .	. . .	. . .	. . .	. . .	. . .	. . .	. . .	. . .	. . .	. . .	. . .	. . .	354
LL37_19	. . .	. . .	. . .	. . .	. . .	. . .	. . .	. . .	. . .	. . .	. . .	. . .	. . .	. . .	. . .	. . .	. . .	. . .	. . .	. . .	. . .	. . .	. . .	. . .	. . .	. . .	. . .	. . .	. . .	. . .	360
LL37_20	. . .	. . .	. . .	. . .	. . .	. . .	. . .	. . .	. . .	. . .	. . .	. . .	. . .	. . .	. . .	. . .	. . .	. . .	. . .	. . .	. . .	. . .	. . .	. . .	. . .	. . .	. . .	. . .	. . .	. . .	360
LL37Iron_1	. . .	. . .	. . .	. . .	. . .	. . .	. . .	. . .	. . .	. . .	. . .	. . .	. . .	. . .	. . .	. . .	. . .	. . .	. . .	. . .	. . .	. . .	. . .	. . .	. . .	. . .	. . .	A . .	. . .	. . .	360
LL37Iron_2	. . .	. . .	. . .	. . .	. . .	. . .	. . .	. . .	. . .	. . .	. . .	. . .	. . .	. . .	. . .	. . .	. . .	. . .	. . .	. . .	. . .	. . .	. . .	. . .	. . .	. . .	. . .	T . .	. . .	. . .	360
LL37Iron_3	. . .	. . .	. . .	. . .	. . .	. . .	. . .	. . .	. . .	. . .	. . .	. . .	. . .	. . .	. . .	. . .	. . .	. . .	. . .	. . .	. . .	. . .	. . .	. . .	. . .	. . .	. . .	. . .	. . .	. . .	360
LL37Iron_4	. . .	. . .	. . .	. . .	. . .	. . .	. . .	. . .	. . .	. . .	. . .	. . .	. . .	. . .	. . .	. . .	. . .	. . .	. . .	. . .	. . .	. . .	. . .	. . .	. . .	. . .	. . .	. . .	. . .	. . .	360
LL37Iron_5	. . .	. . .	. . .	. . .	. . .	. . .	. . .	. . .	. . .	. . .	. . .	. . .	. . .	. . .	. . .	. . .	. . .	. . .	. . .	. . .	. . .	. . .	. . .	. . .	. . .	. . .	. . .	. . .	. . .	. . .	360
LL37Iron_6	. . .	. . .	. . .	. . .	. . .	. . .	. . .	. . .	. . .	. . .	. . .	. . .	. . .	. . .	. . .	. . .	. . .	. . .	. . .	. . .	. . .	. . .	. . .	. . .	. . .	. . .	. . .	. . .	. . .	. . .	360
LL37Iron_7	. . .	. . .	. . .	. . .	. . .	. . .	. . .	. . .	. . .	. . .	. . .	. . .	. . .	. . .	. . .	. . .	. . .	. . .	. . .	. . .	. . .	. . .	. . .	. . .	. . .	. . .	. . .	. . .	. . .	. . .	360
LL37Iron_8	. . .	. . .	. . .	. . .	. . .	. . .	. . .	. . .	. . .	. . .	. . .	. . .	. . .	. . .	. . .	. . .	. . .	. . .	. . .	. . .	. . .	. . .	. . .	. . .	. . .	. . .	. . .	. . .	. . .	. . .	360
LL37Iron_9	. . .	. . .	. . .	. . .	. . .	. . .	. . .	. . .	. . .	. . .	. . .	. . .	. . .	. . .	. . .	. . .	. . .	. . .	. . .	. . .	. . .	. . .	. . .	. . .	. . .	. . .	. . .	. . .	. . .	. . .	360
LL37Iron_10	. . .	. . .	. . .	. . .	. . .	. . .	. . .	. . .	. . .	. . .	. . .	. . .	. . .	. . .	. . .	. . .	. . .	. . .	. . .	. . .	. . .	. . .	. . .	. . .	. . .	. . .	. . .	. . .	. . .	. . .	360
LL37Iron_11	. . .	. . .	. . .	. . .	. . .	. . .	. . .	. . .	. . .	. . .	. . .	. . .	. . .	. . .	. . .	. . .	. . .	. . .	. . .	. . .	. . .	. . .	. . .	. . .	. . .	. . .	. . .	. . .	. . .	. . .	360
LL37Iron_12	. . .	. . .	. . .	. . .	. . .	. . .	. . .	. . .	. . .	. . .	. . .	. . .	. . .	. . .	. . .	. . .	. . .	. . .	. . .	. . .	. . .	. . .	. . .	. . .	. . .	. . .	. . .	. . .	. . .	. . .	360
LL37Iron_13	. . .	. . .	. . .	. . .	. . .	. . .	. . .	. . .	. . .	. . .	. . .	. . .	. . .	. . .	. . .	. . .	. . .	. . .	. . .	. . .	. . .	. . .	. . .	. . .	. . .	. . .	. . .	. . .	. . .	. . .	360
LL37Iron_14	. . .	. . .	. . .	. . .	. . .	. . .	. . .	. . .	. . .	. . .	. . .	. . .	. . .	. . .	. . .	. . .	. . .	. . .	. . .	. . .	. . .	. . .	. . .	. . .	. . .	. . .	. . .	. . .	. . .	. . .	360
LL37Iron_15	. . .	. . .	. . .	. . .	. . .	. . .	. . .	. . .	. . .	. . .	. . .	. . .	. . .	. . .	. . .	. . .	. . .	. . .	. . .	. . .	. . .	. . .	. . .	. . .	. . .	. . .	. . .	. . .	. . .	. . .	360
LL37Iron_16	. . .	. . .	. . .	. . .	. . .	. . .	. . .	. . .	. . .	. . .	. . .	. . .	. . .	. . .	. . .	. . .	. . .	. . .	. . .	. . .	. . .	. . .	. . .	. . .	. . .	. . .	. . .	. . .	. . .	. . .	360
LL37Iron_17	. . .	. . .	. . .	. . .	. . .	. . .	. . .	. . .	. . .	. . .	. . .	. . .	. . .	. . .	. . .	. . .	. . .	. . .	. . .	. . .	. . .	. . .	. . .	. . .	. . .	. . .	. . .	. . .	. . .	. . .	360

```
LL37Iron_18  ... ... ... ... ... ... ... ... ... ... ... ... ... ... ... ... ... ... ... ... ... ... ... ... ... ... ... ... ... ... 359
LL37Iron_19  ... ... ... ... ... ... ... ... ... ... ... ... ... ... ... ... ... ... ... ... ... ... ... ... ... ... ... ... ... ... 360
LL37Iron_20  ... ... ... ... ... ... ... ... ... ... ... ... ... ... ... ... ... ... ... ... ... ... ... ... ... ... ... T.. ... ... 360
```

							380							400						420							440				
WT_PA14_orf	TGG	CCG	CCG	AGC	GGA	CGG	ACC	ATG	GTC	CAC	TGG	TGG	TCG	CAG	AAG	GAG	CGC	GGC	AGC	GTG	GTC	TCG	GTG	TGG	AAC	GTC	GCC	CAC	AAT	GTC	450
iron_1	...	...	...	...	...	...	...	...	...	...	...	...	...	...	...	...	...	...	...	...	...	...	...	...	...	...	...	...	...	...	450
iron_2	...	...	...	...	...	...	...	...	...	...	...	...	...	...	...	...	...	...	...	...	...	...	...	...	...	...	...	...	...	...	444
iron_3	...	...	...	...	...	...	...	...	...	...	...	...	...	...	...	...	...	...	...	...	...	...	...	...	...	...	...	...	...	...	450
iron_4	...	...	...	...	...	...	...	...	...	...	...	...	...	...	...	...	...	...	...	...	...	...	...	...	...	...	...	...	...	...	450
iron_5	...	...	...	...	...	...	...	...	...	...	...	...	...	...	...	...	...	...	...	...	...	...	...	...	...	...	...	...	...	...	450
iron_6	...	...	...	...	...	...	...	...	...	...	...	...	...	...	...	...	...	...	...	...	...	...	...	...	...	...	...	...	...	...	450
iron_7	...	...	...	...	...	...	...	...	...	...	...	...	...	...	...	...	...	...	...	...	...	...	...	...	...	...	...	...	...	...	450
iron_8	...	...	...	...	...	...	...	...	...	...	...	...	...	...	...	...	...	...	...	...	...	...	...	...	...	...	...	...	...	...	450
iron_9	...	...	...	...	...	...	...	...	...	...	...	...	...	...	...	...	...	...	...	...	...	...	...	...	...	...	...	...	...	...	450
iron_10	...	...	...	...	...	...	...	...	...	...	...	...	...	...	...	...	...	...	...	...	...	...	...	...	...	...	...	...	...	...	444
iron_11	...	...	...	...	...	...	...	...	...	...	...	...	...	...	...	...	...	...	...	...	...	...	...	...	...	...	...	...	...	...	444
iron_12	...	...	...	...	...	...	...	...	...	...	...	...	...	...	...	...	...	...	...	...	...	...	...	...	...	...	...	...	...	...	450
iron_13	...	...	...	...	...	...	...	...	...	...	...	...	...	...	...	...	...	...	...	...	...	...	...	...	...	...	...	...	...	...	444
iron_14	...	...	...	...	...	...	...	...	...	...	...	...	...	...	...	...	...	...	...	...	...	...	...	...	...	...	...	...	...	...	444
iron_15	...	...	...	...	...	...	...	...	...	...	...	...	...	...	...	...	...	...	...	...	...	...	...	...	...	...	...	...	...	...	444
iron_16	...	...	...	...	...	...	...	...	...	...	...	...	...	...	...	...	...	...	...	...	...	...	...	...	...	...	...	...	...	...	444
iron_17	...	...	...	...	...	...	...	...	...	...	...	...	...	...	...	...	...	...	...	...	...	...	...	...	...	...	...	...	...	...	444
iron_18	...	...	...	...	...	...	...	...	...	...	...	...	...	...	...	...	...	...	...	...	...	...	...	...	...	...	...	...	...	...	450
iron_19	...	...	...	...	...	...	...	...	...	...	...	...	...	...	...	...	...	...	...	...	...	...	...	...	...	...	...	...	...	...	450
iron_20	...	...	...	...	...	...	...	...	...	...	...	...	...	...	...	...	...	...	...	...	...	...	...	...	...	...	...	...	...	...	450
LL37_1	...	...	...	...	...	...	...	...	...	...	...	...	...	...	...	...	...	...	...	...	...	...	...	...	...	...	...	...	G..	...	450
LL37_2	...	...	...	...	...	...	...	...	...	...	...	...	...	...	...	...	...	...	...	...	...	...	...	...	...	...	...	...	...	...	444
LL37_3	...	...	...	...	...	...	...	...	...	...	...	...	...	...	...	...	...	...	...	...	...	...	...	...	...	...	...	...	...	...	450
LL37_4	...	...	...	...	...	...	...	...	...	...	...	...	...	...	...	...	...	...	...	...	...	...	...	...	...	...	...	...	...	...	450
LL37_5	...	...	...	...	...	...	...	...	...	...	...	...	...	...	...	...	...	...	...	...	...	...	...	...	...	...	...	...	...	...	444
LL37_6	...	...	...	...	...	...	...	...	...	...	...	...	...	...	...	...	...	...	...	...	...	...	...	...	...	...	...	...	...	...	450
LL37_7	...	...	...	...	...	...	...	...	...	...	...	...	...	...	...	...	...	...	...	...	...	...	...	...	...	...	...	...	...	...	444
LL37_8	...	...	...	...	...	...	...	...	...	...	...	...	...	...	...	...	...	...	...	...	...	...	...	...	...	...	...	...	...	...	450
LL37_9	...	...	...	...	...	...	...	...	...	...	...	...	...	...	...	...	...	...	...	...	...	...	...	...	...	...	...	...	...	...	444
LL37_10	...	...	...	...	...	...	...	...	...	...	...	...	...	...	...	...	...	...	...	...	...	...	...	...	...	...	...	...	...	...	450
LL37_11	...	...	...	...	...	...	...	...	...	...	...	...	...	...	...	...	...	...	...	...	...	...	...	...	...	...	...	...	...	...	444
LL37_12	...	...	...	...	...	...	...	...	...	...	...	...	...	...	...	...	...	...	...	...	...	...	...	...	...	...	...	...	...	...	450
LL37_13	...	...	...	...	...	...	...	...	...	...	...	...	...	...	...	A..	...	...	...	...	...	...	...	...	...	...	...	...	...	...	450
LL37_14	...	...	...	...	...	...	...	...	...	...	...	...	...	...	...	...	...	...	...	...	...	...	...	...	...	...	...	...	...	...	450
LL37_15	...	...	...	...	...	...	C..	...	...	...	...	...	...	...	...	...	...	...	...	...	...	...	...	...	...	...	...	...	...	...	450
LL37_16	...	...	...	...	...	...	...	...	...	...	...	...	...	...	...	...	...	...	...	...	...	...	...	...	...	...	...	...	...	...	450
LL37_17	...	...	...	...	...	...	...	...	...	...	...	...	...	...	...	...	...	...	...	...	...	...	...	...	...	...	...	...	...	...	450
LL37_18	...	...	...	...	...	...	...	...	...	...	...	...	...	...	...	...	...	...	...	...	...	...	...	...	...	...	...	...	...	...	444
LL37_19	...	...	...	...	...	...	...	...	...	...	...	...	...	...	...	...	...	...	...	...	...	...	...	...	...	...	...	...	...	...	450
LL37_20	...	...	...	...	...	...	...	...	...	...	...	...	...	...	...	...	...	...	...	...	...	...	...	...	...	...	...	...	...	...	450
LL37Iron_1	...	...	...	...	...	...	...	...	...	...	...	...	...	...	...	...	...	...	...	...	...	...	...	...	...	...	...	...	...	...	450
LL37Iron_2	...	...	...	...	...	...	...	...	...	...	...	...	...	...	...	...	...	...	...	...	...	...	...	...	...	...	...	...	...	...	450
LL37Iron_3	...	...	...	...	...	...	...	...	...	...	...	...	...	...	...	...	...	...	...	...	...	...	...	...	...	...	...	...	...	...	450
LL37Iron_4	...	...	...	...	...	...	...	...	...	...	...	...	...	...	...	...	...	...	...	...	...	...	...	...	...	...	...	...	...	...	450
LL37Iron_5	...	...	...	...	...	...	...	...	...	...	...	...	...	...	...	...	...	...	...	...	...	...	...	...	...	...	...	...	...	...	450
LL37Iron_6	...	...	...	...	...	...	...	...	...	...	...	...	...	...	...	...	...	...	...	...	...	...	...	...	...	...	...	...	...	...	450
LL37Iron_7	...	...	...	...	...	...	...	...	...	...	...	...	...	...	...	...	...	...	...	...	...	...	...	...	...	...	...	...	...	...	450
LL37Iron_8	...	...	...	...	...	...	...	...	...	...	...	...	...	...	...	...	...	...	...	...	...	...	...	...	...	...	...	...	...	...	450
LL37Iron_9	...	...	...	...	...	...	...	...	...	...	...	...	...	...	...	...	...	...	...	...	...	...	...	...	...	...	...	...	...	...	450
LL37Iron_10	...	...	...	...	...	...	...	...	...	...	...	...	...	...	...	...	...	...	...	...	...	...	...	...	...	...	...	...	...	...	450
LL37Iron_11	...	...	...	...	...	...	...	...	...	...	...	...	...	...	...	...	...	...	...	...	...	...	...	...	...	...	.T.	...	...	...	450
LL37Iron_12	...	...	...	...	...	...	...	...	...	...	...	...	...	...	...	...	...	...	...	...	...	...	...	...	...	...	.T.	...	...	...	450
LL37Iron_13	...	...	...	...	...	...	...	...	...	...	...	...	...	...	...	...	...	...	...	...	...	...	...	...	...	...	...	...	...	...	450
LL37Iron_14	...	...	...	...	...	...	...	...	...	...	...	...	...	...	...	...	...	...	...	...	...	...	...	...	...	...	...	...	...	...	450
LL37Iron_15	...	...	...	...	...	...	...	...	...	...	...	...	...	...	...	...	...	...	...	...	...	...	...	...	...	...	...	...	...	...	450
LL37Iron_16	...	...	...	...	...	...	...	...	...	...	...	...	...	...	...	...	...	...	...	...	...	...	...	...	...	...	...	...	...	...	450
LL37Iron_17	...	...	...	...	...	...	...	...	...	...	...	...	...	...	...	...	...	...	...	...	...	...	...	...	...	...	...	...	...	...	450

LL37Iron_18	. . . .	449
LL37Iron_19	. . . .	450
LL37Iron_20	. . . .	450

460 480 500 520 540

WT_PA14_orf	GGC	GGC	GGC	CTG	ATC	GGC	CCG	CTG	TTC	CTG	CTC	GGC	CTG	GCG	CTG	TTC	GGC	GAC	TGG	CGC	TCG	GCC	TTC	TAC	GTG	CCG	GCC	ATC	GTC	GCC	540
iron_1	...	...	...	...	...	...	...	...	...	...	...	...	...	...	...	...	...	...	...	...	...	...	...	...	...	...	...	...	...	...	540
iron_2	...	...	...	...	...	...	...	...	...	...	...	...	...	...	...	...	...	...	...	...	...	...	...	...	...	...	...	...	...	...	534
iron_3	...	...	...	...	...	...	...	...	...	...	...	...	...	...	...	...	...	...	...	...	...	...	...	...	...	...	...	...	...	...	540
iron_4	...	...	...	...	...	...	...	...	...	...	...	...	...	...	...	...	...	...	...	...	...	...	...	...	...	...	...	...	...	...	540
iron_5	...	...	...	...	...	...	...	...	...	...	...	...	...	...	...	...	...	...	...	...	...	...	...	...	...	...	...	...	...	...	540
iron_6	...	...	...	...	...	...	...	...	...	...	...	...	...	...	...	...	...	...	...	...	...	...	...	...	...	...	...	...	...	...	540
iron_7	...	...	...	...	...	...	...	...	...	...	...	...	...	...	...	...	...	...	...	...	...	...	...	...	...	...	...	...	...	...	540
iron_8	...	...	...	...	...	...	...	...	...	...	...	...	...	...	...	...	...	...	...	...	...	...	...	...	...	...	...	...	...	...	540
iron_9	...	...	...	...	...	...	...	...	...	...	...	...	...	...	...	...	...	...	...	...	...	...	...	...	...	...	...	...	...	...	540
iron_10	...	...	...	...	...	...	...	...	...	...	...	...	...	...	...	...	...	...	...	...	...	...	...	...	...	...	...	...	...	...	534
iron_11	...	...	...	...	...	...	...	...	...	...	...	...	...	...	...	...	...	...	...	...	...	...	...	...	...	...	...	...	...	...	534
iron_12	...	...	...	...	...	...	...	...	...	...	...	...	...	...	...	...	...	...	...	...	...	...	...	...	...	...	...	...	...	...	540
iron_13	...	...	...	...	...	...	...	...	...	...	...	...	...	...	...	...	...	...	...	...	...	...	...	...	...	...	...	...	...	...	534
iron_14	...	...	...	...	...	...	...	...	...	...	...	...	...	...	...	...	...	...	...	...	...	...	...	...	...	...	...	...	...	...	534
iron_15	...	...	...	...	...	...	...	...	...	...	...	...	...	...	...	...	...	...	...	...	...	...	...	...	...	...	...	...	...	...	534
iron_16	...	...	...	...	...	...	...	...	...	...	...	...	...	...	...	...	...	...	...	...	...	...	...	...	...	...	...	...	...	...	534
iron_17	...	...	...	...	...	...	...	...	...	...	...	...	...	...	...	...	...	...	...	...	...	...	...	...	...	...	...	...	...	...	534
iron_18	...	...	...	...	...	...	...	...	...	...	...	...	...	...	...	...	...	...	...	...	...	...	...	...	...	...	...	...	...	...	540
iron_19	...	...	...	...	...	...	...	.C.	...	...	...	...	...	...	...	...	...	...	...	...	...	...	...	...	...	...	...	...	...	...	540
iron_20	...	...	...	...	...	...	...	...	...	...	...	...	...	...	...	...	...	...	...	...	...	...	...	...	...	...	...	...	...	...	540
LL37_1	...	...	...	...	...	...	...	...	...	...	...	...	...	...	...	...	...	...	...	...	...	...	...	...	...	...	...	...	...	...	540
LL37_2	...	...	...	...	...	...	...	...	...	...	...	...	...	...	...	...	...	...	...	...	...	...	...	...	...	...	...	...	...	...	534
LL37_3	...	...	...	...	...	...	...	...	...	...	...	...	...	...	...	...	...	...	...	...	...	...	...	...	...	...	...	...	...	...	540
LL37_4	...	...	...	...	...	...	...	...	...	...	...	...	...	...	...	...	...	...	...	...	...	...	...	...	...	...	...	...	...	...	540
LL37_5	...	...	...	...	...	...	...	...	...	...	...	...	...	...	...	...	...	...	...	...	...	...	...	...	...	...	...	...	...	...	534
LL37_6	...	...	...	...	...	...	...	.C.	...	...	...	...	...	...	...	...	...	...	...	...	...	...	...	...	...	...	...	...	...	...	540
LL37_7	...	...	...	...	...	...	...	...	...	...	...	...	...	...	...	...	...	...	...	...	...	...	...	...	...	...	...	...	...	...	534
LL37_8	...	...	...	...	...	...	...	...	...	...	...	...	...	...	...	...	...	...	...	...	...	...	...	...	...	...	...	...	...	...	540
LL37_9	...	...	...	...	...	...	...	...	...	...	...	...	...	...	...	...	...	...	...	...	...	...	...	...	...	...	...	...	...	...	534
LL37_10	...	...	...	...	...	...	...	...	...	...	...	...	...	...	...	...	...	...	...	...	...	...	...	...	...	...	...	...	...	...	540
LL37_11	...	...	...	...	...	...	...	...	...	...	...	...	...	...	...	...	...	...	...	...	...	...	...	...	...	...	...	...	...	...	534
LL37_12	...	...	...	...	...	...	...	...	...	...	...	...	...	...	...	...	...	...	...	...	...	...	...	...	...	...	...	...	...	...	540
LL37_13	...	...	...	...	...	...	...	...	...	...	...	...	...	...	...	...	...	...	...	...	...	...	...	...	...	...	...	...	...	...	540
LL37_14	...	...	...	...	...	...	...	...	...	...	...	...	...	...	...	...	...	...	...	...	...	...	...	...	...	...	...	...	...	...	540
LL37_15	...	...	...	...	...	...	...	...	...	...	...	...	...	...	...	...	...	...	...	...	...	...	...	...	...	...	...	...	...	...	540
LL37_16	...	...	...	...	...	...	...	...	...	...	...	...	...	...	...	...	...	...	...	...	...	...	...	...	...	...	...	...	...	...	540
LL37_17	...	...	...	...	...	...	...	...	...	...	...	...	...	...	...	...	...	...	...	...	...	...	...	...	...	...	...	...	...	...	540
LL37_18	...	...	...	...	...	...	...	...	...	...	...	...	...	...	...	...	...	...	...	...	...	...	...	...	...	...	...	...	...	...	534
LL37_19	...	...	...	...	...	...	...	...	...	...	...	...	...	...	...	...	...	...	...	...	...	...	...	...	...	...	...	...	...	...	540
LL37_20	...	...	...	...	...	...	...	...	...	...	...	...	...	...	...	...	...	...	...	...	...	...	...	...	...	...	...	...	...	...	540
LL37Iron_1	...	...	...	...	...	...	...	...	...	...	...	...	...	...	...	...	...	...	...	...	...	...	...	...	...	...	...	...	...	...	540
LL37Iron_2	...	...	...	...	...	...	...	...	...	...	...	...	...	...	...	...	...	...	...	...	...	...	...	...	...	...	...	...	...	...	540
LL37Iron_3	...	...	...	...	...	...	...	...	...	...	...	...	...	...	...	...	...	...	...	...	...	...	...	...	...	...	...	...	...	...	540
LL37Iron_4	...	...	...	...	...	...	...	...	...	...	...	...	...	...	...	...	...	...	...	...	...	...	...	...	...	...	...	...	...	...	540
LL37Iron_5	...	...	...	...	...	...	...	...	...	...	...	...	...	...	...	...	...	...	...	...	...	...	...	...	...	...	...	...	...	...	540
LL37Iron_6	...	...	...	...	...	...	...	...	...	...	...	...	...	...	...	...	...	...	...	...	...	...	...	...	...	...	...	...	...	...	540
LL37Iron_7	...	...	...	...	...	...	...	...	...	...	...	...	...	...	...	...	...	...	...	...	...	...	...	...	...	...	...	...	...	...	540
LL37Iron_8	...	...	...	...	...	...	...	...	...	...	...	...	...	...	...	...	...	...	...	...	...	...	...	...	...	...	...	...	...	...	540
LL37Iron_9	...	...	...	...	...	...	...	...	...	...	...	...	...	...	...	...	...	...	...	...	...	...	...	...	...	...	...	...	...	...	540
LL37Iron_10	...	...	...	...	...	...	...	...	...	...	...	...	...	...	...	...	...	...	...	...	...	...	...	...	...	...	...	...	...	...	540
LL37Iron_11	...	...	...	...	...	...	...	...	...	...	...	...	...	...	...	...	...	...	...	...	...	...	...	...	...	...	...	...	...	...	540
LL37Iron_12	...	...	...	...	...	...	...	...	...	...	...	...	...	...	...	...	...	...	...	...	...	...	...	...	...	...	...	...	...	...	540
LL37Iron_13	...	...	...	...	...	...	...	...	...	...	...	...	...	...	...	...	...	...	...	...	...	...	...	...	...	...	...	...	...	...	540
LL37Iron_14	...	...	...	...	...	...	...	...	...	...	...	...	...	...	...	...	...	...	...	...	...	...	...	...	...	...	...	...	...	...	540
LL37Iron_15	...	...	...	...	...	...	...	...	...	...	...	...	...	...	...	...	...	...	...	...	...	...	...	...	...	...	...	...	...	...	540
LL37Iron_16	...	...	...	...	...	...	...	...	...	...	...	...	...	...	...	...	...	...	...	...	...	...	...	...	...	...	...	...	...	...	540
LL37Iron_17	...	...	...	...	...	...	...	...	...	...	...	...	...	...	...	...	...	...	...	...	...	...	...	...	...	...	...	...	...	...	540

```
LL37Iron_18  ... ... ... ... ... ... ... ... ... ... ... ... ... ... ... ... ... ... ... ... ... ... ... ... ... ... ... ... ... ... 539
LL37Iron_19  ... ... ... ... ... ... ... ... ... ... ... ... ... ... ... ... ... ... ... ... ... ... ... ... ... ... ... ... ... ... 540
LL37Iron_20  ... ... ... ... ... ... ... ... ... ... ... ... ... ... ... ... ... ... ... ... ... ... ... ... ... ... ... ... ... ... 540
```

```
                                                   560                                     580                                   600                                     620
                                                    |                                       |                                     |                                       |
WT_PA14_orf  GTG GGC GTA GCC GTG TTC GCC TAT TTC GCC ATG CGC GAC ACC CCG CAG TCC TGC GGC CTG CCG CCG ATC GAG AAG CAT AAG GAC GAC TAC 630
     iron_1  ... ... ... ... ... ... ... ... ... ... ... ... ... ... ... ... ... ... ... ... ... ... ... ... ... ... ... ... ... ... 630
     iron_2  ... ... ... ... ... ... ... ... ... ... ... ... ... ... ... ... ... ... ... ... ... ... ... ... ... ... ... ... ... ... 624
     iron_3  ... ... ... ... ... ... ... ... ... ... ... ... ... ... ... ... ... ... ... ... ... ... ... ... ... ... ... ... ... ... 630
     iron_4  ... ... ... ... ... ... ... ... ... ... ... ... ... ... ... ... ... ... ... ... ... ... ... ... ... ... ... ... ... ... 630
     iron_5  ... ... ... ... ... ... ... ... ... ... ... ... ... ... ... ... ... ... ... ... ... ... ... ... ... ... ... ... ... ... 630
     iron_6  ... ... ... ... ... ... ... ... ... ... ... ... ... ... ... ... ... ... ... ... ... ... ... ... ... ... ... ... ... ... 630
     iron_7  ... ... ... ... ... ... ... ... ... ... ... ... ... ... ... ... ... ... ... ... ... ... ... ... ... ... ... ... ... ... 630
     iron_8  ... ... ... ... ... ... ... ... ... ... ... ... ... ... ... ... ... ... ... ... ... ... ... ... ... ... ... ... ... ... 630
     iron_9  ... ... ... ... ... ... ... ... ... ... ... ... ... ... ... ... ... ... ... ... ... ... ... ... ... ... ... ... ... ... 630
    iron_10  ... ... ... ... ... ... ... ... ... ... ... ... ... ... ... ... ... ... ... ... ... ... ... ... ... ... ... ... ... ... 624
    iron_11  ... ... ... ... ... ... ... ... ... ... ... ... ... ... ... ... ... ... ... ... ... ... ... ... ... ... ... ... ... ... 624
    iron_12  ... ... ... ... ... ... ... ... ... ... ... ... ... ... ... ... ... ... ... ... ... ... ... ... ... ... ... ... ... ... 630
    iron_13  ... ... ... ... ... ... ... ... ... ... ... ... ... ... ... ... ... ... ... ... ... ... ... ... ... ... ... ... ... ... 624
    iron_14  ... ... ... ... ... ... ... ... ... ... ... ... ... ... ... ... ... ... ... ... ... ... ... ... ... ... ... ... ... ... 624
    iron_15  ... ... ... ... ... ... ... ... ... ... ... ... ... ... ... ... ... ... ... ... ... ... ... ... ... ... ... ... ... ... 624
    iron_16  ... ... ... ... ... ... ... ... ... ... ... ... ... ... ... ... ... ... ... ... ... ... ... ... ... ... ... ... ... ... 624
    iron_17  ... ... ... ... ... ... ... ... ... ... ... ... ... ... ... ... ... ... ... ... ... ... ... ... ... ... ... ... ... ... 624
    iron_18  ... ... ... ... ... ... ... ... ... ... ... ... ... ... ... ... ... ... ... ... ... ... ... ... ... ... ... ... ... ... 630
    iron_19  ... ... ... ... ... ... ... ... ... ... ... ... ... ... ... ... ... ... ... ... ... ... ... ... ... ... ... ... ... ... 630
    iron_20  ... ... ... ... ... ... ... ... ... ... ... ... ... ... ... ... ... ... .A. ... ... ... ... ... ... ... ... ... ... ... 630
     LL37_1  ... ... ... ... ... ... ... ... ... ... ... ... ... ... ... ... ... ... ... ... ... ... ... ... ... ... ... ... ... ... 630
     LL37_2  ... ... ... ... ... ... ... ... ... ... ... ... ... ... ... ... ... ... ... ... ... ... ... ... ... ... ... ... ... ... 624
     LL37_3  ... ... ... ... ... ... ... ... ... ... ... ... ... ... ... ... ... ... ... ... ... ... ... ... ... ... ... ... ... ... 630
     LL37_4  ... ... ... ... ... ... ... ... ... ... ... ... ... ... ... ... ... ... ... ... ... ... ... ... ... ... ... ... ... ... 630
     LL37_5  ... ... ... ... ... ... ... ... ... ... ... ... ... ... ... ... ... ... ... ... ... ... ... ... ... ... ... ... ... ... 624
     LL37_6  ... ... ... ... ... ... ... ... ... ... ... ... ... ... ... ... ... ... ... ... ... ... ... ... ... ... ... ... ... ... 630
     LL37_7  ... ... ... ... ... ... ... ... ... ... ... ... ... ... ... ... ... ... ... ... ... ... ... ... ... ... ... ... ... ... 624
     LL37_8  ... ... ... ... ... ... ... ... ... ... ... ... ... ... ... ... ... ... ... ... ... ... ... ... ... ... ... ... ... ... 630
     LL37_9  ... ... ... ... ... ... ... ... ... ... ... ... ... ... ... ... ... ... ... ... ... ... ... ... ... ... ... ... ... ... 624
    LL37_10  ... ... ... ... ... ... ... ... ... ... ... ... ... ... ... ... ... ... ... ... ... ... ... ... ... ... ... ... ... ... 630
    LL37_11  ... ... ... ... ... ... ... ... ... ... ... ... ... ... ... ... ... ... ... ... ... ... ... ... ... ... ... ... ... ... 624
    LL37_12  ... ... ... ... ... ... ... ... ... ... ... ... ... ... ... ... ... ... ... ... ... ... ... ... ... ... ... ... ... ... 630
    LL37_13  ... ... ... ... ... ... ... ... ... ... ... ... ... ... ... ... ... ... ... ... ... ... ... ... ... ... ... ... ... ... 630
    LL37_14  ... ... ... ... ... ... ... ... ... ... ... ... ... ... ... ... ... ... ... ... ... ... ... ... ... ... ... ... ... ... 630
    LL37_15  ... ... ... ... ... ... ... ... ... ... ... ... ... ... ... ... ... ... ... ... ... ... ... ... ... ... ... ... ... ... 630
    LL37_16  ... ... ... ... ... ... ... ... ... ... ... ... ... ... ... ... ... ... ... ... ... ... ... ... ... ... ... ... ... ... 630
    LL37_17  ... ... ... ... ... ... ... ... ... ... ... ... ... ... ... ... ... ... ... ... ... ... ... ... ... ... ... ... ... ... 630
    LL37_18  ... ... ... ... ... ... ... ... ... ... ... ... ... ... ... ... ... ... ... ... ... ... ... ... ... ... ... ... ... ... 624
    LL37_19  ... ... ... ... ... ... ... ... ... ... ... ... ... ... ... ... ... ... ... ... ... ... ... ... ... ... ... ... ... ... 630
    LL37_20  ... ... ... ... ... ... ... ... ... ... ... ... ... ... ... ... ... ... ... ... ... ... ... ... ... ... ... ... ... ... 630
 LL37Iron_1  ... ... ... ... ... ... ... ... ... ... ... ... ... ... ... ... ... ... ... ... ... ... ... ... ... ... ... ... ... ... 630
 LL37Iron_2  ... ... ... ... ... ... ... ... ... ... ... ... ... ... ... ... ... ... ... ... ... ... ... ... ... ... ... ... ... ... 630
 LL37Iron_3  ... ... ... ... ... ... ... ... ... ... ... ... ... ... ... ... ... ... ... ... ... ... ... ... ... ... ... ... ... ... 630
 LL37Iron_4  ... ... ... ... ... ... ... ... ... ... ... ... ... ... ... ... ... ... ... ... ... ... ... ... ... ... ... ... ... ... 630
 LL37Iron_5  ... ... ... ... ... ... ... ... ... ... ... ... ... ... ... ... ... ... ... ... ... ... ... ... ... ... ... ... ... ... 630
 LL37Iron_6  ... ... ... ... ... ... ... ... ... ... ... ... ... ... ... ... ... ... ... ... ... ... ... ... ... ... ... ... ... ... 630
 LL37Iron_7  ... ... ... ... ... ... ... ... ... ... ... ... ... ... ... ... ... ... ... ... ... ... ... ... ... ... ... ... ... ... 630
 LL37Iron_8  ... ... ... ... ... ... ... ... ... ... ... ... ... ... ... ... ... ... ... ... ... ... ... ... ... ... ... ... ... ... 630
 LL37Iron_9  ... ... ... ... ... ... ... ... ... ... ... ... ... ... ... ... ... ... ... ... ... ... ... ... ... ... ... ... ... ... 630
LL37Iron_10  ... ... ... ... ... ... ... ... ... ... ... ... ... ... ... ... ... ... ... ... ... ... ... ... ... ... ... ... ... ... 630
LL37Iron_11  ... ... ... ... ... ... ... ... ... ... ... ... ... ... ... ... ... ... ... ... ... ... ... ... ... ... ... ... ... ... 630
LL37Iron_12  ... ... ... ... ... ... ... ... ... ... ... ... ... ... ... ... ... ... ... ... ... ... ... ... ... ... ... ... ... ... 630
LL37Iron_13  ... ... ... ... ... ... ... ... ... ... ... ... ... ... ... ... ... ... ... ... ... ... ... ... ... ... ... ... ... ... 630
LL37Iron_14  ... ... ... ... ... ... ... ... ... ... ... ... ... ... ... ... ... ... ... ... ... ... ... ... ... ... ... ... ... ... 630
LL37Iron_15  ... ... ... ... ... ... ... ... ... ... ... ... ... ... ... ... ... ... ... ... ... ... ... ... ... ... ... ... ... ... 630
LL37Iron_16  ... ... ... ... ... ... ... ... ... ... ... ... ... ... ... ... ... ... ... ... ... ... ... ... ... ... ... ... ... ... 630
LL37Iron_17  ... ... ... ... ... ... ... ... ... ... ... ... ... ... ... ... ... ... ... ... ... ... ... ... ... ... ... ... ... ... 630
```

LL37Iron_18 629
LL37Iron_19 630
LL37Iron_20 630

				640						660							680							700						720	
WT_PA14_orf	CCC	GAA	GGC	TAC	GAC	GAA	CAC	CAC	GAG	CGC	GAG	CTG	TCG	ACC	AAG	GAG	ATC	TTC	GTC	ACC	TAC	GTG	CTG	CGC	AAC	AAG	CTG	CTC	TGG	TAC	720
iron_1	...	...	...	...	...	...	...	...	...	...	...	...	...	...	...	...	...	...	...	...	...	...	...	...	...	...	...	...	...	...	720
iron_2	...	...	...	...	...	...	...	...	...	...	...	...	...	...	...	...	...	...	...	...	...	...	...	...	...	...	...	...	...	...	714
iron_3	...	...	...	...	...	...	...	...	...	...	...	...	...	...	...	...	...	...	...	...	...	...	...	...	...	...	...	...	...	...	720
iron_4	...	...	...	...	...	...	...	...	...	...	...	...	...	...	...	...	...	...	...	...	...	...	...	...	...	...	...	...	...	...	720
iron_5	...	...	...	...	...	...	...	...	...	...	...	...	...	...	...	...	...	...	...	...	...	...	...	...	...	...	...	...	...	...	720
iron_6	...	...	...	...	...	...	...	...	...	...	...	...	...	...	...	...	...	...	...	...	...	...	...	...	...	...	...	...	...	...	720
iron_7	...	...	...	...	...	...	...	...	...	...	...	...	...	...	...	...	...	...	...	...	...	...	...	...	...	...	...	...	...	...	720
iron_8	...	...	...	...	...	...	...	...	...	...	...	...	...	...	...	...	...	...	...	...	...	...	...	...	...	...	...	...	...	...	720
iron_9	...	...	...	...	...	...	...	...	...	...	...	...	...	...	...	...	...	...	...	...	...	...	...	...	...	...	...	...	...	...	720
iron_10	...	...	...	...	...	...	...	...	...	...	...	...	...	...	...	...	...	...	...	...	...	...	...	...	...	...	...	...	...	...	714
iron_11	...	...	...	...	...	...	...	...	...	...	...	...	...	...	...	...	...	...	...	...	...	...	...	...	...	...	...	...	...	...	714
iron_12	...	...	...	...	...	...	...	...	...	...	...	...	...	...	...	...	...	...	...	...	...	...	...	...	...	...	...	...	...	...	720
iron_13	...	...	...	...	...	...	...	...	...	...	...	...	...	...	...	...	...	...	...	...	...	...	...	...	...	...	...	...	...	...	714
iron_14	...	...	...	...	...	...	...	...	...	...	...	...	...	...	...	...	...	...	...	...	...	...	...	...	...	...	...	...	...	...	714
iron_15	...	...	...	...	...	...	...	...	...	...	...	...	...	...	...	...	...	...	...	...	...	...	...	...	...	...	...	...	...	...	714
iron_16	...	...	...	...	...	...	...	...	...	...	...	...	...	...	...	...	...	...	...	...	...	...	...	...	...	...	...	...	...	...	714
iron_17	...	...	...	...	...	...	...	...	...	...	...	...	...	...	...	...	...	...	...	...	...	...	...	...	...	...	...	...	...	...	714
iron_18	...	...	...	...	...	...	...	...	...	...	...	...	...	...	...	...	...	...	...	...	...	...	...	...	...	...	...	...	...	...	720
iron_19	...	...	...	...	...	...	...	...	...	...	...	...	...	...	...	...	...	...	...	...	...	...	...	...	...	...	...	...	...	...	720
iron_20	...	...	...	...	...	...	...	...	...	...	...	...	...	...	...	...	...	...	...	...	...	...	...	...	...	...	...	...	...	...	720
LL37_1	...	...	...	...	...	...	...	...	...	...	...	...	...	...	...	...	...	...	...	...	...	...	...	...	...	...	...	...	...	...	720
LL37_2	...	...	...	...	...	...	...	...	...	...	...	...	...	...	...	...	...	...	...	...	...	...	...	...	...	...	...	...	...	...	714
LL37_3	...	...	...	...	...	...	...	...	...	...	...	...	...	...	...	...	...	...	...	...	...	...	...	...	...	...	...	...	...	...	720
LL37_4	...	...	...	...	...	...	...	...	...	...	...	...	...	...	...	...	...	...	...	...	...	...	...	...	...	...	...	...	...	...	720
LL37_5	...	...	...	...	...	...	...	...	...	...	...	...	...	...	...	...	...	...	...	...	...	...	...	...	...	...	...	...	...	...	714
LL37_6	...	...	...	...	...	...	...	...	...	...	...	...	...	...	...	...	...	...	...	...	...	...	...	...	...	...	...	...	...	...	720
LL37_7	...	...	...	...	...	...	...	...	...	...	...	...	...	...	...	...	...	...	...	...	...	...	...	...	...	...	...	...	...	...	714
LL37_8	...	...	...	...	...	...	...	...	...	...	...	...	...	...	...	...	...	...	...	...	...	...	...	...	...	...	...	...	...	...	720
LL37_9	...	...	...	...	...	...	...	...	...	...	...	...	...	...	...	...	...	...	...	...	...	...	...	...	...	...	...	...	...	...	714
LL37_10	...	...	...	...	...	...	...	...	...	...	...	...	...	...	...	...	...	...	...	...	...	...	...	...	...	...	...	...	...	...	720
LL37_11	...	...	...	...	...	...	...	...	...	...	...	...	...	...	...	...	...	...	...	...	...	...	...	...	...	...	...	...	...	...	714
LL37_12	...	...	...	...	...	...	...	...	...	...	...	...	...	...	...	...	...	...	...	...	...	...	...	...	...	...	...	...	...	...	720
LL37_13	...	...	...	...	...	...	...	...	...	...	...	...	...	...	...	...	...	...	...	...	...	...	...	...	...	...	...	...	...	...	720
LL37_14	...	...	...	...	...	...	...	...	...	...	...	...	...	...	...	...	...	...	...	...	...	...	...	...	...	...	...	...	...	...	720
LL37_15	...	...	...	...	...	...	...	...	...	...	...	...	...	...	...	...	...	...	...	...	...	...	...	...	...	...	...	...	...	...	720
LL37_16	...	...	...	...	...	...	...	...	...	...	...	...	...	...	...	...	...	...	...	...	...	...	...	...	...	...	...	...	...	...	720
LL37_17	...	...	...	...	...	...	...	...	...	...	...	...	...	...	...	...	...	...	...	...	...	...	...	...	...	...	...	...	...	...	720
LL37_18	...	...	...	...	...	...	...	...	...	...	...	...	...	...	...	...	...	...	...	...	...	...	...	...	...	...	...	...	...	...	714
LL37_19	...	...	...	...	...	...	...	...	...	...	...	...	...	...	...	...	...	...	...	...	...	...	...	...	...	...	...	...	...	...	720
LL37_20	...	...	...	...	...	...	...	...	...	...	...	...	...	...	...	...	...	...	...	...	...	...	...	...	...	...	...	...	...	...	720
LL37Iron_1	...	...	...	...	...	...	...	...	...	...	...	...	...	...	...	...	...	...	...	...	...	...	...	...	...	...	...	...	...	...	720
LL37Iron_2	...	...	...	...	...	...	...	...	...	...	...	...	...	...	...	...	...	...	...	...	...	...	...	...	...	...	...	...	...	...	720
LL37Iron_3	...	...	...	...	...	...	...	...	...	...	...	...	...	...	...	...	...	...	...	...	...	...	...	...	...	...	...	...	...	...	720
LL37Iron_4	...	...	...	...	...	...	...	...	...	...	...	...	...	...	...	...	...	...	...	...	...	...	...	...	...	...	...	...	...	...	720
LL37Iron_5	...	...	...	...	...	...	...	...	...	...	...	...	...	...	...	...	...	...	...	...	...	...	...	...	...	...	...	...	...	...	720
LL37Iron_6	...	...	...	...	...	...	...	...	...	...	...	...	...	...	...	...	...	...	...	...	...	...	...	...	...	...	...	...	...	...	720
LL37Iron_7	...	...	...	...	...	...	...	...	...	...	...	...	...	...	...	...	...	...	...	...	...	...	...	...	...	...	...	...	...	...	720
LL37Iron_8	...	...	...	...	...	...	...	...	...	...	...	...	...	...	...	...	...	...	...	...	...	...	...	...	...	...	...	...	...	...	720
LL37Iron_9	...	...	...	...	...	...	...	...	...	...	...	...	...	...	...	...	...	...	...	...	...	...	...	...	...	...	...	...	...	...	720
LL37Iron_10	...	...	...	...	...	...	...	...	...	...	...	...	...	...	...	...	...	...	...	...	...	...	...	...	...	...	...	...	...	...	720
LL37Iron_11	...	...	...	...	...	...	...	...	...	...	...	...	...	...	...	...	...	...	...	...	...	...	...	...	...	...	...	...	...	...	720
LL37Iron_12	...	...	...	...	...	...	...	...	...	...	...	...	...	...	...	...	...	...	...	...	...	...	...	...	...	...	...	...	...	...	720
LL37Iron_13	...	...	...	...	...	...	...	...	...	...	...	...	...	...	...	...	...	...	...	...	...	...	...	...	...	...	...	...	...	...	720
LL37Iron_14	...	...	...	...	...	...	...	...	...	...	...	...	...	...	...	...	...	...	...	...	...	...	...	...	...	...	...	...	...	...	720
LL37Iron_15	...	...	...	...	...	...	...	...	...	...	...	...	...	...	...	...	...	...	...	...	...	...	...	...	...	...	...	...	...	...	720
LL37Iron_16	...	...	...	...	...	...	...	...	...	...	...	...	...	...	...	...	...	...	...	...	...	...	...	...	...	...	...	...	...	...	720
LL37Iron_17	...	...	...	...	...	...	...	...	...	...	...	...	...	...	...	...	...	...	...	...	...	...	...	...	...	...	...	...	...	...	720

```
LL37Iron_18  ... ... ... ... ... ... ... ... ... ... ... ... ... ... ... ... ... ... ... ... ... ... ... ... ... ... ... ... ... ... 719
LL37Iron_19  ... ... ... ... ... ... ... ... ... ... ... ... ... ... ... ... ... ... ... ... ... ... ... ... ... ... ... ... ... ... 720
LL37Iron_20  ... ... ... ... ... ... ... ... ... ... ... ... ... ... ... ... ... ... ... ... ... ... ... ... ... ... ... ... ... ... 720
```

						740							760						780							800				
WT_PA14_orf	ATC	GCC	CTG	GCC	AAC	GTG	TTC	GTC	TAC	CTG	CTG	CGC	TAC	GGT	GTG	CTC	GAC	TGG	GCG	CCG	ACC	TAC	CTC	AAG	GAG	GCC	AAG	CAC	TTC	AAC	810
iron_1	...	...	...	...	...	...	...	...	...	...	...	...	...	...	...	...	...	...	...	...	...	...	...	...	...	...	...	...	...	...	810
iron_2	...	...	...	...	...	...	...	...	...	...	...	...	...	...	...	...	...	...	...	...	...	...	...	...	...	...	...	...	...	...	804
iron_3	...	...	...	...	...	...	...	...	...	...	...	...	...	...	...	...	...	...	...	...	...	...	...	...	.T.	...	...	...	...	...	810
iron_4	...	...	...	...	...	...	...	...	...	...	...	...	...	...	...	...	...	...	...	...	...	...	...	...	...	...	...	...	...	...	810
iron_5	...	...	...	...	...	...	...	...	...	...	...	...	...	...	...	...	...	...	...	...	...	...	...	...	.T.	...	...	...	...	...	810
iron_6	...	...	...	...	...	...	...	...	...	...	...	...	...	...	...	...	...	...	...	...	...	...	...	...	...	...	...	...	...	...	810
iron_7	...	...	...	...	...	...	...	...	...	...	...	...	...	...	...	...	...	...	...	...	...	...	...	...	...	...	...	...	...	...	810
iron_8	...	...	...	...	...	...	...	...	...	...	...	...	...	...	...	...	...	...	...	...	...	...	...	...	...	...	...	...	...	...	810
iron_9	...	...	...	...	...	...	...	...	...	...	...	...	...	...	...	...	...	A..	...	...	...	...	...	...	...	...	...	...	...	...	810
iron_10	...	...	...	...	...	...	...	...	...	...	...	...	...	...	...	...	...	...	...	...	...	...	...	...	...	...	...	...	...	...	804
iron_11	...	...	...	...	...	...	...	...	...	...	...	...	...	...	...	...	...	...	...	...	...	...	...	...	...	...	...	...	...	...	804
iron_12	...	...	...	...	...	...	...	...	...	...	...	...	...	...	...	...	...	...	...	...	...	...	...	...	...	...	...	...	...	...	810
iron_13	...	...	...	...	...	...	...	...	...	...	...	...	...	...	...	...	...	...	...	...	...	...	...	...	...	...	...	...	...	...	804
iron_14	...	...	...	...	...	...	...	...	...	...	...	...	...	...	...	...	...	...	...	...	...	...	...	...	...	...	...	...	...	...	804
iron_15	...	...	...	...	...	...	...	...	...	...	...	...	...	...	...	...	...	...	...	...	...	...	...	...	...	...	...	...	...	...	804
iron_16	...	...	...	...	...	...	...	...	...	...	...	...	...	...	...	...	...	...	...	...	...	...	...	...	...	...	...	...	...	...	804
iron_17	...	...	...	...	...	...	...	...	...	...	...	...	...	...	...	...	...	...	...	...	...	...	...	...	...	...	...	...	...	...	804
iron_18	...	...	...	...	...	...	...	...	...	...	...	...	...	...	...	...	...	...	...	...	...	...	...	...	...	...	...	...	...	...	810
iron_19	...	...	...	...	...	...	...	...	...	...	...	...	...	...	...	...	...	...	...	...	...	...	...	...	...	...	...	...	...	...	810
iron_20	...	...	...	...	...	...	...	...	...	...	...	...	...	...	...	...	...	...	...	...	...	...	...	...	...	...	...	...	...	...	810
LL37_1	...	...	...	...	...	...	...	...	...	...	...	...	...	...	...	...	...	...	...	...	...	...	...	...	...	...	...	...	...	...	810
LL37_2	...	...	...	...	...	...	...	...	...	...	...	...	...	...	...	...	...	...	...	...	...	...	...	...	...	...	...	...	...	...	804
LL37_3	...	...	...	...	...	...	...	...	...	...	...	...	...	...	...	...	...	...	...	...	...	...	...	...	...	...	...	...	...	...	810
LL37_4	...	...	...	...	...	...	...	...	...	...	...	...	...	...	...	...	...	...	...	...	...	...	...	...	...	...	...	...	...	...	810
LL37_5	...	...	...	...	...	...	...	...	...	...	...	...	...	...	...	...	...	...	...	...	...	...	...	...	...	...	...	...	...	...	804
LL37_6	...	...	...	...	...	...	...	...	...	...	...	...	...	...	...	...	...	...	...	...	...	...	...	...	...	...	...	...	...	...	810
LL37_7	...	...	...	...	...	...	...	...	...	...	...	...	...	...	...	...	...	...	...	...	...	...	...	...	...	...	...	...	...	...	804
LL37_8	...	...	...	...	...	...	...	...	...	...	...	...	...	...	...	...	...	...	...	...	...	...	...	...	...	...	...	...	...	...	810
LL37_9	...	...	...	...	...	...	...	...	...	...	...	...	...	...	...	...	...	...	...	...	...	...	...	...	...	...	...	...	...	...	804
LL37_10	...	...	...	...	...	...	...	...	...	...	...	...	...	...	...	...	...	...	...	...	...	...	...	...	...	...	...	...	...	...	810
LL37_11	...	...	...	...	...	...	...	...	...	...	...	...	...	...	...	...	...	...	...	...	...	...	...	...	...	...	...	...	...	...	804
LL37_12	...	...	...	...	...	...	...	...	...	...	...	...	...	...	...	...	...	...	...	...	...	...	...	...	...	...	...	...	...	...	810
LL37_13	...	...	...	...	...	...	...	...	...	...	...	...	...	...	...	...	...	...	...	...	...	...	...	...	...	...	...	...	...	...	810
LL37_14	...	...	...	...	...	...	...	...	...	...	...	...	...	...	...	...	...	...	...	...	...	...	...	...	...	...	...	...	...	...	810
LL37_15	...	...	...	...	...	...	...	...	...	...	...	...	...	...	...	...	...	...	...	...	...	...	...	...	...	...	...	...	...	...	810
LL37_16	...	...	...	...	...	...	...	...	...	...	...	...	...	...	...	...	...	...	...	...	...	...	...	...	...	...	...	...	...	...	810
LL37_17	...	...	...	...	...	...	...	...	...	...	...	...	...	...	...	...	...	.A.	...	...	...	...	...	...	...	...	...	...	...	...	810
LL37_18	...	...	...	...	...	...	...	...	...	...	...	...	...	...	...	...	...	...	...	...	...	...	...	...	...	...	...	...	...	...	804
LL37_19	...	...	...	...	...	...	...	...	...	...	...	...	...	...	...	...	...	...	...	...	...	...	...	...	...	...	...	...	...	...	810
LL37_20	...	...	...	...	...	...	...	...	...	...	...	...	...	...	...	...	...	...	...	...	...	...	...	...	...	...	...	...	...	...	810
LL37Iron_1	...	...	...	...	...	...	...	...	...	...	...	...	...	...	...	...	...	...	...	...	...	...	...	...	...	...	...	...	...	...	810
LL37Iron_2	...	...	...	...	...	...	...	...	...	...	...	...	...	...	...	...	...	...	...	...	...	...	...	...	...	...	...	...	...	...	810
LL37Iron_3	...	...	...	...	...	...	...	...	...	...	...	...	...	...	...	...	..G	...	...	...	...	...	...	...	...	...	...	...	...	...	810
LL37Iron_4	...	...	...	...	...	...	...	...	...	...	...	...	...	...	...	...	...	...	...	...	...	...	...	...	...	...	...	...	...	...	810
LL37Iron_5	...	...	...	...	...	...	...	...	...	...	...	...	...	...	...	...	...	...	...	...	...	...	...	...	...	...	...	...	...	...	810
LL37Iron_6	...	...	...	...	...	...	...	...	...	...	...	...	...	...	...	...	...	...	...	...	...	...	...	...	...	...	...	...	...	...	810
LL37Iron_7	...	...	...	...	...	...	...	...	...	...	...	...	...	...	...	...	...	...	...	...	...	...	...	...	...	...	...	...	...	...	810
LL37Iron_8	...	...	...	...	...	...	...	...	...	...	...	...	...	...	...	...	...	...	...	...	...	...	...	...	...	...	...	...	...	...	810
LL37Iron_9	...	...	...	...	...	...	...	...	...	...	...	...	...	...	...	...	...	...	...	...	...	...	...	...	...	...	...	...	...	...	810
LL37Iron_10	...	...	...	...	...	...	...	...	...	...	...	...	...	...	...	...	...	...	...	...	...	...	...	...	...	...	...	...	...	...	810
LL37Iron_11	...	...	...	...	...	...	...	...	...	...	...	...	...	...	...	...	...	...	...	...	...	...	...	...	...	...	...	...	...	...	810
LL37Iron_12	...	...	...	...	...	...	...	...	...	...	...	...	...	...	...	...	...	...	...	...	...	...	...	...	...	...	...	...	...	...	810
LL37Iron_13	...	...	...	...	...	...	...	...	...	...	...	...	...	...	...	...	..G	...	...	...	...	...	...	...	...	...	...	...	...	...	810
LL37Iron_14	...	...	...	...	...	...	...	...	...	...	...	...	...	...	...	...	...	...	...	...	...	...	...	...	...	...	...	...	...	...	810
LL37Iron_15	...	...	...	...	...	...	...	...	...	...	...	...	...	...	...	...	...	...	...	...	...	...	...	...	...	...	...	...	...	...	810
LL37Iron_16	...	...	...	...	...	...	...	...	...	...	...	...	...	...	...	...	...	...	...	...	...	...	...	...	...	...	...	...	...	...	810
LL37Iron_17	...	...	...	...	...	...	...	...	...	...	...	...	...	...	...	...	...	...	...	...	...	...	...	...	...	...	...	...	...	...	810

```
LL37Iron_18 ... ... ... ... ... ... ... ... ... ... ... ... ... ... ... ... ... ... ... ... ... ... ... ... ... ... ... ... ... ... 809
LL37Iron_19 ... ... ... ... ... ... ... ... ... ... ... ... ... ... ... ... ... ... ... ... ... ... ... ... ... ... ... ... ... ... 810
LL37Iron_20 ... ... ... ... ... ... ... ... ... ... ... ... ... ... ... ... ... ... ... ... ... ... ... ... ... ... ... ... ... ... 810
```

				820						840							860							880						900	
WT_PA14_orf	GTC	GAC	AAG	TCG	TCC	TGG	GCG	TAC	TTC	TTC	TAC	GAA	TGG	GCG	GGG	ATT	CCC	GGC	ACC	CTG	CTC	TGC	GGC	TGG	CTG	TCG	GAC	AAG	CTG	TTC	900
iron_1	...	...	...	...	...	...	...	...	...	...	...	...	...	...	...	...	...	...	...	...	...	...	...	...	...	...	...	...	...	...	900
iron_2	...	...	...	...	...	...	...	...	...	...	...	...	...	...	...	...	...	...	...	...	...	...	...	...	...	...	...	...	...	...	894
iron_3	...	...	...	...	...	...	...	...	...	...	...	...	...	...	...	...	...	...	...	...	...	...	...	...	...	...	...	...	...	...	900
iron_4	...	...	...	...	...	...	...	...	...	...	...	...	...	...	...	...	...	...	...	...	...	...	...	...	...	...	...	...	...	...	900
iron_5	...	...	...	...	...	...	...	...	...	...	...	...	...	...	...	...	...	...	...	...	...	...	...	...	...	...	...	...	...	...	900
iron_6	...	...	...	...	...	...	...	...	...	...	...	...	...	...	...	...	...	...	...	...	...	...	...	...	...	...	...	...	...	...	900
iron_7	...	...	...	...	...	...	...	...	...	...	...	...	...	...	...	...	...	...	...	...	...	...	...	...	...	...	...	...	...	...	900
iron_8	...	...	...	...	...	...	...	...	...	...	...	...	...	...	...	.C.	...	...	...	...	...	...	...	...	...	...	...	...	...	...	900
iron_9	...	...	...	...	...	...	...	...	...	...	...	...	...	...	...	...	...	...	...	...	...	...	...	...	...	...	...	...	...	...	900
iron_10	...	...	...	...	...	...	...	...	...	...	...	...	...	...	...	...	...	...	...	...	...	...	...	...	...	...	...	...	...	...	894
iron_11	...	...	...	...	...	...	...	...	...	...	...	...	...	...	...	...	...	...	...	...	...	...	...	...	...	...	...	...	...	...	894
iron_12	...	...	...	...	...	...	...	...	...	...	...	...	...	...	...	...	...	...	...	...	...	...	...	...	...	...	...	...	...	...	900
iron_13	...	...	...	...	...	...	...	...	...	...	...	...	...	...	...	...	...	...	...	...	...	...	...	...	...	...	...	...	...	...	894
iron_14	...	...	...	...	...	...	...	...	...	...	...	...	...	...	...	...	...	...	...	...	...	...	...	...	...	...	...	...	...	...	894
iron_15	...	...	...	...	...	...	...	...	...	...	...	...	...	...	...	...	...	...	...	...	...	...	...	...	...	...	...	...	...	...	894
iron_16	...	...	...	...	...	...	...	...	...	...	...	...	...	...	...	...	...	...	...	...	...	...	...	...	...	...	...	...	...	...	894
iron_17	...	...	...	...	...	...	...	...	...	...	...	...	...	...	...	...	...	...	...	...	...	...	...	...	...	...	...	...	...	...	894
iron_18	...	...	...	...	...	...	...	...	...	...	...	...	...	...	...	...	...	...	...	...	...	...	...	...	...	...	...	...	...	...	900
iron_19	...	...	...	...	...	...	...	...	...	...	...	...	...	...	...	...	...	...	...	...	...	...	...	...	...	...	...	...	...	...	900
iron_20	...	...	...	...	...	...	...	...	...	...	...	...	...	...	...	...	...	...	...	...	...	...	...	...	...	...	...	...	...	...	900
LL37_1	...	...	...	...	...	...	...	...	...	...	...	...	...	...	...	...	...	...	...	...	...	...	...	...	...	...	...	...	...	...	900
LL37_2	...	...	...	...	...	...	...	...	...	...	...	...	...	...	...	...	...	...	...	...	...	...	...	...	...	...	...	...	...	...	894
LL37_3	...	...	...	...	...	...	...	...	...	...	...	...	...	...	...	...	...	...	...	...	...	...	...	...	...	...	...	...	...	...	900
LL37_4	...	...	...	...	...	...	...	...	...	...	...	...	...	...	...	...	...	...	...	...	...	...	...	...	...	...	...	...	...	...	900
LL37_5	...	...	...	...	...	...	...	...	...	...	...	...	...	...	...	...	...	...	...	...	...	...	...	...	...	...	...	...	...	...	894
LL37_6	...	...	...	...	...	...	...	...	...	...	...	...	...	...	...	...	...	...	...	...	...	...	...	...	...	...	...	...	...	...	900
LL37_7	...	...	...	...	...	...	...	...	...	...	...	...	...	...	...	...	...	...	...	...	...	...	...	...	...	...	...	...	...	...	894
LL37_8	...	...	...	...	...	...	...	...	...	...	..A	...	...	...	...	...	...	...	...	...	...	...	...	...	...	...	...	...	...	...	900
LL37_9	...	...	...	...	...	...	...	...	...	...	...	...	...	...	...	...	...	...	...	...	...	...	...	...	...	...	...	...	...	...	894
LL37_10	...	...	...	...	...	...	...	...	...	...	...	...	...	...	...	...	...	...	...	...	...	...	...	...	...	...	...	...	...	...	900
LL37_11	...	...	...	...	...	...	...	...	...	...	...	...	...	...	...	...	...	...	...	...	...	...	...	...	...	...	...	...	...	...	894
LL37_12	...	...	...	...	...	...	...	...	...	...	...	...	...	...	...	...	...	...	...	...	...	...	...	...	...	...	...	...	...	...	900
LL37_13	...	...	...	...	...	...	...	...	...	...	...	...	...	...	...	...	...	...	...	...	...	...	...	...	...	...	...	...	...	...	900
LL37_14	...	...	...	...	...	...	...	...	...	...	...	...	...	...	...	...	...	...	...	...	...	...	...	...	...	...	...	...	...	...	900
LL37_15	...	...	...	...	...	...	...	...	...	...	...	...	...	...	...	...	...	...	...	...	...	...	...	...	...	...	...	...	...	...	900
LL37_16	...	...	...	...	...	...	...	...	...	...	...	...	...	...	...	...	...	...	...	...	...	...	...	...	...	...	...	...	...	...	900
LL37_17	...	...	...	...	...	...	...	...	...	...	...	...	...	...	...	...	...	...	...	...	...	...	...	...	...	...	...	...	...	...	900
LL37_18	...	...	...	...	...	...	...	...	...	...	...	...	...	...	...	...	...	...	...	...	...	...	...	...	...	...	...	...	...	...	894
LL37_19	...	...	...	...	...	...	...	...	...	...	...	...	...	...	...	...	...	A..	...	...	...	...	...	...	...	...	...	...	...	...	900
LL37_20	...	...	...	...	...	...	...	...	...	...	...	...	...	...	...	...	...	...	...	...	...	...	...	...	...	...	...	...	...	...	900
LL37Iron_1	...	...	...	...	...	...	...	...	...	...	...	...	...	...	...	...	...	...	...	...	...	...	...	...	...	...	...	...	...	...	900
LL37Iron_2	...	...	...	...	...	...	...	...	...	...	...	...	...	...	...	...	...	...	...	...	...	...	...	...	...	...	...	...	...	...	900
LL37Iron_3	...	...	...	...	...	...	...	...	...	...	...	...	...	...	...	...	...	...	...	...	...	...	...	...	...	...	...	...	...	...	900
LL37Iron_4	...	...	...	...	...	...	...	...	...	...	...	...	...	...	...	...	...	...	...	...	...	...	...	...	...	...	...	...	...	...	900
LL37Iron_5	...	...	...	...	...	...	...	...	...	...	...	...	...	...	...	...	...	...	...	...	...	...	...	...	...	...	...	...	...	...	900
LL37Iron_6	...	...	...	...	...	...	...	...	...	...	...	...	...	...	...	...	...	...	...	...	...	...	...	...	...	...	...	...	...	...	900
LL37Iron_7	...	...	...	...	...	...	...	...	...	...	...	...	...	...	...	...	...	...	...	...	...	...	...	...	...	...	...	...	...	...	900
LL37Iron_8	...	...	...	...	...	...	...	...	...	...	...	...	...	...	...	...	...	...	...	...	...	...	...	...	...	...	...	...	...	...	900
LL37Iron_9	...	...	...	...	...	...	...	...	...	...	...	...	...	...	...	...	...	...	...	...	...	...	...	...	...	...	...	...	...	...	900
LL37Iron_10	...	...	...	...	...	...	...	...	...	...	...	...	...	...	...	...	...	...	...	...	...	...	...	...	...	...	...	...	...	...	900
LL37Iron_11	...	...	...	...	...	...	...	...	...	...	...	...	...	...	...	...	...	...	...	...	...	...	...	...	...	...	...	...	...	...	900
LL37Iron_12	...	...	...	...	...	...	...	...	...	...	...	...	...	...	...	...	...	...	...	...	...	...	...	...	...	...	...	...	...	...	900
LL37Iron_13	...	...	...	...	...	...	...	...	...	...	...	...	...	...	...	...	...	...	...	...	...	...	...	...	...	...	...	...	...	...	900
LL37Iron_14	...	...	...	...	...	...	...	...	...	...	...	...	...	...	...	...	...	...	...	...	...	...	...	...	...	...	...	...	...	...	900
LL37Iron_15	...	...	...	...	...	...	...	...	...	...	...	...	...	...	...	...	...	...	...	...	...	...	...	...	...	...	...	...	...	...	900
LL37Iron_16	...	...	...	...	...	...	...	...	...	...	...	...	...	...	...	...	...	...	...	...	...	...	...	...	...	...	...	...	...	...	900
LL37Iron_17	...	...	...	...	...	...	...	...	...	...	...	...	...	...	...	...	...	...	...	...	...	...	...	...	...	...	...	...	...	...	900

LL37Iron_18	... ...	899
LL37Iron_19	... ...	900
LL37Iron_20	... ...	900

							920							940						960							980				
WT_PA14_orf	AAG	GGC	AAT	CGC	GGC	GCC	ACC	GGC	GTG	GTG	TTC	ATG	ATC	CTG	GTG	ACC	GTC	GGC	ACC	CTG	ATC	TAC	TGG	CTG	AAC	CCG	GCC	GGC	AAC	CCG	990
iron_1	. . .	. . .	. . .	. . .	. . .	. . .	. . .	. . .	. . .	. . .	. . .	. . .	. . .	. . .	. . .	. . .	. . .	. . .	. . .	. . .	. . .	. . .	. . .	. . .	. . .	. . .	. . .	. . .	. . .	. . .	990
iron_2	. . .	. . .	. . .	. . .	. . .	. . .	. . .	. . .	. . .	. . .	. . .	. . .	. . .	. . .	. . .	. . .	. . .	. . .	. . .	. . .	. . .	. . .	. . .	. . .	. . .	. . .	. . .	. . .	. . .	. . .	984
iron_3	. . .	. . .	. . .	. . .	. . .	. . .	. . .	. . .	. . .	. . .	. . .	. . .	. . .	. . .	. . .	. . .	. . .	. . .	. . .	. . .	. . .	. . .	. . .	. . .	. . .	. . .	. . .	. . .	. . .	. . .	990
iron_4	. . .	. . .	. . .	. . .	. . .	. . .	. . .	. . .	. . .	. . .	. . .	. . .	. . .	. . .	. . .	. . .	. . .	. . .	. . .	. . .	. . .	. . .	. . .	. . .	. . .	. . .	. . .	. . .	. . .	. . .	990
iron_5	. . .	. . .	. . .	. . .	. . .	. . .	. . .	. . .	. . .	. . .	. . .	. . .	. . .	. . .	. . .	. . .	. . .	. . .	. . .	. . .	. . .	. . .	. . .	. . .	. . .	. . .	. . .	. . .	. . .	. . .	990
iron_6	. . .	. . .	. . .	. . .	. . .	. . .	. . .	. . .	. . .	. . .	. . .	. . .	. . .	. . .	. . .	. . .	. . .	. . .	. . .	. . .	. . .	. . .	. . .	. . .	. . .	. . .	. . .	. . .	. . .	. . .	990
iron_7	. . .	. . .	. . .	. . .	. . .	. . .	. . .	. . .	. . .	. . .	. . .	. . .	. . .	. . .	. . .	. . .	. . .	. . .	. . .	. . .	. . .	. . .	. . .	. . .	. . .	. . .	. . .	. . .	. . .	. . .	990
iron_8	. . .	. . .	. . .	. . .	. . .	. . .	. . .	. . .	. . .	. . .	. . .	. . .	. . .	. . .	. . .	. . .	. . .	. . .	. . .	. . .	. . .	. . .	. . .	. . .	. . .	. . .	. . .	. . .	. . .	. . .	990
iron_9	. . .	. . .	. . .	. . .	. . .	. . .	. . .	. . .	. . .	. . .	. . .	. . .	. . .	. . .	. . .	. . .	. . .	. . .	. . .	. . .	. . .	. . .	. . .	. . .	. . .	. . .	. . .	. . .	. . .	. . .	990
iron_10	. . .	. . .	. . .	. . .	. . .	. . .	. . .	. . .	. . .	. . .	. . .	. . .	. . .	. . .	. . .	. . .	. . .	. . .	. . .	. . .	. . .	. . .	. . .	. . .	. . .	. . .	. . .	. . .	. . .	. . .	984
iron_11	. . .	. . .	. . .	. . .	. . .	. . .	. . .	. . .	. . .	. . .	. . .	. . .	. . .	. . .	. . .	. . .	. . .	. . .	. . .	. . .	. . .	. . .	. . .	. . .	. . .	. . .	. . .	. . .	. . .	. . .	984
iron_12	. . .	. . .	. . .	. . .	. . .	. . .	. . .	. . .	. . .	. . .	. . .	. . .	. . .	. . .	. . .	. . .	. . .	. . .	. . .	. . .	. . .	. . .	. . .	. . .	. . .	. . .	. . .	. . .	. . .	. . .	990
iron_13	. . .	. . .	. . .	. . .	. . .	. . .	. . .	. . .	. . .	. . .	. . .	. . .	. . .	. . .	. . .	. . .	. . .	. . .	. . .	. . .	. . .	. . .	. . .	. . .	. . .	. . .	. . .	. . .	. . .	. . .	984
iron_14	. . .	. . .	. . .	. . .	. . .	. . .	. . .	. . .	. . .	. . .	. . .	. . .	. . .	. . .	. . .	. . .	. . .	. . .	. . .	. . .	. . .	. . .	. . .	. . .	. . .	. . .	. . .	. . .	. . .	. . .	984
iron_15	. . .	. . .	. . .	. . .	. . .	. . .	. . .	. . .	. . .	. . .	. . .	. . .	. . .	. . .	. . .	. . .	. . .	. . .	. . .	. . .	. . .	. . .	. . .	. . .	. . .	. . .	. . .	. . .	. . .	. . .	984
iron_16	. . .	. . .	. . .	. . .	. . .	. . .	. . .	. . .	. . .	. . .	. . .	. . .	. . .	. . .	. . .	. . .	. . .	. . .	. . .	. . .	. . .	. . .	. . .	. . .	. . .	. . .	. . .	. . .	. . .	. . .	984
iron_17	. . .	. . .	. . .	. . .	. . .	. . .	. . .	. . .	. . .	. . .	. . .	. . .	. . .	. . .	. . .	. . .	. . .	. . .	. . .	. . .	. . .	. . .	. . .	. . .	. . .	. . .	. . .	. . .	. . .	. . .	984
iron_18	. . .	. . .	. . .	. . .	. . .	. . .	. . .	. . .	. . .	. . .	. . .	. . .	. . .	. . .	. . .	. . .	. . .	. . .	. . .	. . .	. . .	. . .	. . .	. . .	. . .	. . .	. . .	. . .	. . .	. . .	990
iron_19	. . .	. . .	. . .	. . .	. . .	. . .	. . .	. . .	. . .	. . .	. . .	. . .	. . .	. . .	. . .	. . .	. . .	. . .	. . .	. . .	. . .	. . .	. . .	. . .	. . .	. . .	. . .	. . .	. . .	. . .	990
iron_20	. . .	. . .	. . .	. . .	. . .	. . .	. . .	. . .	. . .	. . .	. . .	. . .	. . .	. . .	. . .	. . .	. . .	. . .	. . .	. . .	. . .	. . .	. . .	. . .	. . .	. . .	. . .	. . .	. . .	. . .	990
LL37_1	. . .	. . .	. . .	. . .	. . .	. . .	. . .	. . .	. . .	. . .	. . .	. . .	. . .	. . .	. . .	. . .	. . .	. . .	. . .	. . .	. . .	. . .	. . .	. . .	. . .	. . .	. . .	. . .	. . .	. . .	990
LL37_2	. . .	. . .	. . .	. . .	. . .	. . .	. . .	. . .	. . .	. . .	. . .	. . .	. . .	. . .	. . .	. . .	. . .	. . .	. . .	. . .	. . .	. . .	. . .	. . .	. . .	. . .	. . .	. . .	. . .	. . .	984
LL37_3	. . .	. . .	. . .	. . .	. . .	. . .	. . .	. . .	. . .	. . .	. . .	. . .	. . .	. . .	. . .	. . .	. . .	. . .	C . .	. . .	. . .	. . .	. . .	. . .	. . .	. . .	. . .	. . .	. . .	. . .	990
LL37_4	. . .	. . .	. . .	. . .	. . .	. . .	. . .	. . .	. . .	. . .	. . .	. . .	. . .	. . .	. . .	. . .	. . .	. . .	. . .	. . .	. . .	. . .	. . .	. . .	. . .	. . .	. . .	. . .	. . .	. . .	990
LL37_5	. . .	. . .	. . .	. . .	. . .	. . .	. . .	. . .	. . .	. . .	. . .	. . .	. . .	. . .	. . .	. . .	. . .	. . .	. . .	. . .	. . .	. . .	. . .	. . .	. . .	. . .	. . .	. . .	. . .	. . .	984
LL37_6	. . .	. . .	. . .	. . .	. . .	. . .	. . .	. . .	. . .	. . .	. . .	. . .	. . .	. . .	. . .	. . .	. . .	. . .	. . .	. . .	. . .	. . .	. . .	. . .	. . .	. . .	. . .	. . .	. . .	. . .	990
LL37_7	. . .	. . .	. . .	. . .	. . .	. . .	. . .	. . .	. . .	. . .	. . .	. . .	. . .	. . .	. . .	. . .	. . .	. . .	. . .	. . .	. . .	. . .	. . .	. . .	. . .	. . .	. . .	. . .	. . .	. . .	984
LL37_8	. . .	. . .	. . .	. . .	. . .	. . .	. . .	. . .	. . .	. . .	. . .	. . .	. . .	. . .	. . .	. . .	. . .	. . .	. . .	. . .	. . .	. . .	. . .	. . .	. . .	. . .	. . .	. . .	. . .	. . .	990
LL37_9	. . .	. . .	. . .	. . .	. . .	. . .	. . .	. . .	. . .	. . .	. . .	. . .	. . .	. . .	. . .	. . .	. . .	. . .	. . .	. . .	. . .	. . .	. . .	. . .	. . .	. . .	. . .	. . .	. . .	. . .	984
LL37_10	. . .	. . .	. . .	. . .	. . .	. . .	. . .	. . .	. . .	. . .	. . .	. . .	. . .	. . .	. . .	. . .	. . .	. . .	. . .	. . .	. . .	. . .	. . .	. . .	. . .	. . .	. . .	. . .	. . .	. . .	990
LL37_11	. . .	. . .	. . .	. . .	. . .	. . .	. . .	. . .	. . .	. . .	. . .	. . .	. . .	. . .	. . .	. . .	. . .	. . .	. . .	. . .	. . .	. . .	. . .	. . .	. . .	. . .	. . .	. . .	. . .	. . .	984
LL37_12	. . .	. . .	. . .	. . .	. . .	. . .	. . .	. . .	. . .	. . .	. . .	. . .	. . .	. . .	C . .	. . .	. . .	. . .	. . .	. . .	. . .	. . .	. . .	. . .	. . .	. . .	. . .	. . .	. . .	. . .	990
LL37_13	. . .	. . .	. . .	. . .	. . .	. . .	. . .	. . .	. . .	. . .	. . .	. . .	. . .	. . .	. . .	. . .	. . .	. . .	. . .	. . .	. . .	. . .	. . .	. . .	. . .	. . .	. . .	. . .	. . .	. . .	990
LL37_14	. . .	. . .	. . .	. . .	. . .	. . .	. . .	. . .	. . .	. . .	. . .	. . .	. . .	. . .	. . .	. . .	. . .	. . .	. . .	. . .	. . .	. . .	. . .	. . .	. . .	. . .	. . .	. . .	. . .	. . .	990
LL37_15	. . .	. . .	. . .	. . .	. . .	. . .	. . .	. . .	. . .	. . .	. . .	. . .	. . .	. . .	. . .	. . .	. . .	. . .	. . .	. . .	. . .	. . .	. . .	. . .	. . .	. . .	. . .	. . .	. . .	. . .	990
LL37_16	. . .	. . .	. . .	. . .	. . .	. . .	. . .	. . .	. . .	. . .	. . .	. . .	. . .	. . .	. . .	. . .	. . .	. . .	. . .	. . .	. . .	. . .	. . .	. . .	. . .	. . .	. . .	. . .	. . .	. . .	990
LL37_17	. . .	. . .	. . .	. . .	. . .	. . .	. . .	. . .	. . .	. . .	. . .	. . .	. . .	. . .	. . .	. . .	. . .	. . .	. . .	. . .	. . .	. . .	. . .	. . .	. . .	. . .	. . .	. . .	. . .	. . .	990
LL37_18	. . .	. . .	. . .	. . .	. . .	. . .	. . .	. . .	. . .	. . .	. . .	. . .	. . .	. . .	. . .	. . .	. . .	. . .	. . .	. . .	. . .	. . .	. . .	. . .	. . .	. . .	. . .	. . .	. . .	. . .	984
LL37_19	. . .	. . .	. . .	. . .	. . .	. . .	. . .	. . .	. . .	. . .	. . .	. . .	. . .	. . .	. . .	. . .	. . .	. . .	. . .	. . .	. . .	. . .	. . .	. . .	. . .	. . .	. . .	. . .	. . .	. . .	990
LL37_20	. . .	. . .	. . .	. . .	. . .	. . .	. . .	. . .	. . .	. . .	. . .	. . .	. . .	. . .	. . .	. . .	. . .	. . .	. . .	. . .	. . .	. . .	. . .	. . .	. . .	. . .	. . .	. . .	. . .	. . .	990
LL37Iron_1	. . .	. . .	. . .	. . .	. . .	. . .	. . .	. . .	. . .	. . .	. . .	. . .	. . .	. . .	. . .	. . .	. . .	. . .	. . .	. . .	. . .	. . .	. . .	. . .	. . .	. . .	. . .	. . .	. . .	. . .	990
LL37Iron_2	. . .	. . .	. . .	. . .	. . .	. . .	. . .	. . .	. . .	. . .	. . .	. . .	. . .	. . .	. . .	. . .	. . .	. . .	. . .	. . .	. . .	. . .	. . .	. . .	. . .	. . .	. . .	. . .	. . .	. . .	990
LL37Iron_3	. . .	. . .	. . .	. . .	. . .	. . .	. . .	. . .	. . .	. . .	. . .	. . .	. . .	. . .	. . .	. . .	. . .	. . .	. . .	. . .	. . .	. . .	. . .	. . .	. . .	. . .	. . .	. . .	. . .	. . .	990
LL37Iron_4	. . .	. . .	. . .	. . .	. . .	. . .	. . .	. . .	. . .	. . .	. . .	. . .	. . .	. . .	. . .	. . .	. . .	. . .	. . .	. . .	. . .	. . .	. . .	. . .	. . .	. . .	. . .	. . .	. . .	. . .	990
LL37Iron_5	. . .	. . .	. . .	. . .	. . .	. . .	. . .	. . .	. . .	. . .	. . .	. . .	. . .	. . .	. . .	. . .	. . .	. . .	. . .	. . .	. . .	. . .	. . .	. . .	. . .	. . .	. . .	. . .	. . .	. . .	990
LL37Iron_6	. . .	. . .	. . .	. . .	. . .	. . .	. . .	. . .	. . .	. . .	. . .	. . .	. . .	. . .	. . .	. . .	. . .	. . .	. . .	. . .	. . .	. . .	. . .	. . .	. . .	. . .	. . .	. . .	. . .	. . .	990
LL37Iron_7	. . .	. . .	. . .	. . .	. . .	. . .	. . .	. . .	. . .	. . .	. . .	. . .	. . .	. . .	. . .	. . .	. . .	. . .	. . .	. . .	. . .	. . .	. . .	. . .	. . .	. . .	. . .	. . .	. . .	. . .	990
LL37Iron_8	. . .	. . .	. . .	. . .	. . .	. . .	. . .	. . .	. . .	. . .	. . .	. . .	. . .	. . .	. . .	. . .	. . .	. . .	. . .	. . .	. . .	. . .	. . .	. . .	. . .	. . .	. . .	. . .	. . .	. . .	990
LL37Iron_9	. . .	. . .	. . .	. . .	. . .	. . .	. . .	. . .	. . .	. . .	. . .	. . .	. . .	. . .	. . .	. . .	. . .	. . .	. . .	. . .	. . .	. . .	. . .	. . .	. . .	. . .	. . .	. . .	. . .	. . .	990
LL37Iron_10	. . .	. . .	. . .	. . .	. . .	. . .	. . .	. . .	. . .	. . .	. . .	. . .	. . .	. . .	. . .	. . .	. . .	. . .	. . .	. . .	. . .	. . .	. . .	. . .	. . .	. . .	. . .	. . .	. . .	. . .	990
LL37Iron_11	. . .	. . .	. . .	. . .	. . .	. . .	. . .	. . .	. . .	. . .	. . .	. . .	. . .	. . .	. . .	. . .	. . .	. . .	. . .	. . .	. . .	. . .	. . .	. . .	. . .	. . .	. . .	. . .	. . .	. . .	990
LL37Iron_12	. . .	. . .	. . .	. . .	. . .	. . .	. . .	. . .	. . .	. . .	. . .	. . .	. . .	. . .	. . .	. . .	. . .	. . .	. . .	. . .	. . .	. . .	. . .	. . .	. . .	. . .	. . .	. . .	. . .	. . .	990
LL37Iron_13	. . .	. . .	. . .	. . .	. . .	. . .	. . .	. . .	. . .	. . .	. . .	. . .	. . .	. . .	. . .	. . .	. . .	. . .	. . .	. . .	. . .	. . .	. . .	. . .	. . .	. . .	. . .	. . .	. . .	. . .	990
LL37Iron_14	. . .	. . .	. . .	. . .	. . .	. . .	. . .	. . .	. . .	. . .	. . .	. . .	. . .	. . .	. . .	. . .	. . .	. . .	. . .	. . .	. . .	. . .	. . .	. . .	. . .	. . .	. . .	. . .	. . .	. . .	990
LL37Iron_15	. . .	. . .	. . .	. . .	. . .	. . .	. . .	. . .	. . .	. . .	. . .	. . .	. . .	. . .	. . .	. . .	. . .	. . .	. . .	. . .	. . .	. . .	. . .	. . .	. . .	. . .	. . .	. . .	. . .	. . .	990
LL37Iron_16	. . .	. . .	. . .	. . .	. . .	. . .	. . .	. . .	. . .	. . .	. . .	. . .	. . .	. . .	. . .	. . .	. . .	. . .	. . .	. . .	. . .	. . .	. . .	. . .	. . .	. . .	. . .	. . .	. . .	. . .	990
LL37Iron_17	. . .	. . .	. . .	. . .	. . .	. . .	. . .	. . .	. . .	. . .	. . .	. . .	. . .	. . .	. . .	. . .	. . .	. . .	. . .	. . .	. . .	. . .	. . .	. . .	. . .	. . .	. . .	. . .	. . .	. . .	990

LL37Iron_18 989
LL37Iron_19 990
LL37Iron_20 990

				1.000						1.020							1.040							1.060						1.080	
WT_PA14_orf	GCG	ATC	GAC	ATG	GCC	GCG	CTG	ATC	ATG	ATC	GGC	TTC	CTG	ATC	TAC	GGC	CCG	GTG	ATG	CTG	ATC	GGC	TTG	CAG	GCC	CTG	GAG	CTG	GCG	CCG	1080
iron_1	. . .	. . .	. . .	. . .	. . .	. . .	. . .	. . .	. . .	. . .	. . .	. . .	. . .	. . .	. . .	. . .	. . .	. . .	. . .	. . .	. . .	. . .	. . .	. . .	. . .	. . .	. . .	. . .	. . .	. . .	1080
iron_2	. . .	. . .	. . .	. . .	. . .	. . .	. . .	. . .	. . .	. . .	. . .	. . .	. . .	. . .	. . .	. . .	. . .	. . .	. . .	. . .	. . .	. . .	. . .	. . .	. . .	. . .	. . .	. . .	. . .	. . .	1074
iron_3	. . .	. . .	. . .	. . .	. . .	. . .	. . .	. . .	. . .	. . .	. . .	. . .	. . .	. . .	. . .	. . .	. . .	. . .	. . .	. . .	. . .	. . .	. . .	. . .	. . .	. . .	. . .	. . .	. . .	. . .	1080
iron_4	. . .	. . .	. . .	. . .	. . .	. . .	. . .	. . .	. . .	. . .	. . .	. . .	. . .	. . .	. . .	. . .	. . .	. . .	. . .	. . .	. . .	. . .	. . .	. . .	. . .	. . .	. . .	. . .	. . .	. . .	1080
iron_5	. . .	. . .	. . .	. . .	. . .	. . .	. . .	. . .	. . .	. . .	. . .	. . .	. . .	. . .	. . .	. . .	. . .	. . .	. . .	. . .	. . .	. . .	. . .	. . .	. . .	. . .	. . .	. . .	. . .	. . .	1080
iron_6	. . .	. . .	. . .	. . .	. . .	. . .	. . .	. . .	. . .	. . .	. . .	. . .	. . .	. . .	. . .	. . .	. . .	. . .	. . .	. . .	. . .	. . .	. . .	. . .	. . .	. . .	. . .	. . .	. . .	. . .	1080
iron_7	. . .	. . .	. . .	. . .	. . .	. . .	. . .	. . .	. . .	. . .	. . .	. . .	. . .	. . .	. . .	. . .	. . .	. . .	. . .	. . .	. . .	. . .	. . .	. . .	. . .	. . .	. . .	. . .	. . .	. . .	1080
iron_8	. . .	. . .	. . .	. . .	. . .	. . .	. . .	. . .	. . .	. . .	. . .	. . .	. . .	. . .	. . .	. . .	. . .	. . .	. . .	. . .	. . .	. . .	. . .	. . .	. . .	. . .	. . .	. . .	. . .	. . .	1080
iron_9	. . .	. . .	. . .	. . .	. . .	. . .	. . .	. . .	. . .	. . .	. . .	. . .	. . .	. . .	. . .	. . .	. . .	. . .	. . .	. . .	. . .	. . .	. . .	. . .	. . .	. . .	. . .	. . .	. . .	. . .	1080
iron_10	. . .	. . .	. . .	. . .	. . .	. . .	. . .	. . .	. . .	. . .	. . .	. . .	. . .	. . .	. . .	. . .	. . .	. . .	. . .	. . .	. . .	. . .	. . .	. . .	. . .	. . .	. . .	. . .	. . .	. . .	1074
iron_11	. . .	. . .	. . .	. . .	. . .	. . .	. . .	. . .	. . .	. . .	. . .	. . .	. . .	. . .	. . .	. . .	. . .	. . .	. . .	. . .	. . .	. . .	. . .	. . .	. . .	. . .	. . .	. . .	. . .	. . .	1074
iron_12	. . .	. . .	. . .	. . .	. . .	. . .	. . .	. . .	. . .	. . .	. . .	. . .	. . .	. . .	. . .	. . .	. . .	. . .	. . .	. . .	. . .	. . .	. . .	. . .	. . .	. . .	. . .	. . .	. . .	. . .	1080
iron_13	. . .	. . .	. . .	. . .	. . .	. . .	. . .	. . .	. . .	. . .	. . .	. . .	. . .	. . .	. . .	. . .	. . .	. . .	. . .	. . .	. . .	. . .	. . .	. . .	. . .	. . .	. . .	. . .	. . .	. . .	1074
iron_14	. . .	. . .	. . .	. . .	. . .	. . .	. . .	. . .	. . .	. . .	. . .	. . .	. . .	. . .	. . .	. . .	. . .	. . .	. . .	. . .	. . .	. . .	. . .	. . .	. . .	. . .	. . .	. . .	. . .	. . .	1074
iron_15	. . .	. . .	. . .	. . .	. . .	. . .	. . .	. . .	. . .	. . .	. . .	. . .	. . .	. . .	. . .	. . .	. . .	. . .	. . .	. . .	. . .	. . .	. . .	. . .	. . .	. . .	. . .	. . .	. . .	. . .	1074
iron_16	. . .	. . .	. . .	. . .	. . .	. . .	. . .	. . .	. . .	. . .	. . .	. . .	. . .	. . .	. . .	. . .	. . .	. . .	. . .	. . .	. . .	. . .	. . .	. . .	. . .	. . .	. . .	. . .	. . .	. . .	1074
iron_17	. . .	. . .	. . .	. . .	. . .	. . .	. . .	. . .	. . .	. . .	. . .	. . .	. . .	. . .	. . .	. . .	. . .	. . .	. . .	. . .	. . .	. . .	. . .	. . .	. . .	. . .	. . .	. . .	. . .	. . .	1074
iron_18	. . .	. . .	. . .	. . .	. . .	. . .	. . .	. . .	. . .	. . .	. . .	. . .	. . .	. . .	. . .	. . .	. . .	. . .	. . .	. . .	. . .	. . .	. . .	. . .	. . .	. . .	. . .	. . .	. . .	. . .	1080
iron_19	. . .	. . .	. . .	. . .	. . .	. . .	. . .	. . .	. . .	. . .	. . .	. . .	. . .	. . .	. . .	. . .	. . .	. . .	. . .	. . .	. . .	. . .	. . .	. . .	. . .	. . .	. . .	. . .	. . .	. . .	1080
iron_20	. . .	. . .	. . .	. . .	. . .	. . .	. . .	. . .	. . .	. . .	. . .	. . .	. . .	. . .	. . .	. . .	. . .	. . .	. . .	. . .	. . .	. . .	. . .	. . .	. . .	. . .	. . .	. . .	. . .	. . .	1080
LL37_1	. . .	. . .	. . .	. . .	. . .	. . .	. . .	. . .	. . .	. . .	. . .	. . .	. . .	. . .	. . .	. . .	. . .	. . .	. . .	. . .	. . .	. . .	. . .	. . .	. . .	. . .	. . .	. . .	. . .	. . .	1080
LL37_2	. . .	. . .	. . .	. . .	. . .	. . .	. . .	. . .	. . .	. . .	. . .	. . .	. . .	. . .	. . .	. . .	. . .	. . .	. . .	. . .	. . .	. . .	. . .	. . .	. . .	. . .	. . .	. . .	. . .	. . .	1074
LL37_3	. . .	. . .	. . .	. . .	. . .	. . .	. . .	. . .	. . .	. . .	. . .	. . .	. . .	. . .	. . .	. . .	. . .	. . .	. . .	. . .	. . .	. . .	. . .	. . .	. . .	. . .	. . .	. . .	. . .	. . .	1080
LL37_4	. . .	. . .	. . .	. . .	. . .	. . .	. . .	. . .	. . .	. . .	. . .	. . .	. . .	. . .	. . .	. . .	. . .	. . .	. . .	. . .	. . .	. . .	. . .	. . .	. . .	. . .	. . .	. . .	. . .	. . .	1080
LL37_5	. . .	. . .	. . .	. . .	. . .	. . .	. . .	. . .	. . .	. . .	. . .	. . .	. . .	. . .	. . .	. . .	. . .	. . .	. . .	. . .	. . .	. . .	. . .	. . .	. . .	. . .	. . .	. . .	. . .	. . .	1074
LL37_6	. . .	. . .	. . .	. . .	. . .	. . .	. . .	. . .	. . .	. . .	. . .	. . .	. . .	. . .	. . .	. . .	. . .	. . .	. . .	. . .	. . .	. . .	. . .	. . .	. . .	. . .	. . .	. . .	. . .	. . .	1080
LL37_7	. . .	. . .	. . .	. . .	. . .	. . .	. . .	. . .	. . .	. . .	. . .	. . .	. . .	. . .	. . .	. . .	. . .	. . .	. . .	. . .	. . .	. . .	. . .	. . .	. . .	. . .	. . .	. . .	. . .	. . .	1074
LL37_8	. . .	. . .	. . .	. . .	. . .	. . .	. . .	. . .	. . .	. . .	. . .	. . .	. . .	. . .	. . .	. . .	. . .	. . .	. . .	. . .	. . .	. . .	. . .	. . .	. . .	. . .	. . .	. . .	. . .	. . .	1080
LL37_9	. . .	. . .	. . .	. . .	. . .	. . .	. . .	. . .	. . .	. . .	. . .	. . .	. . .	. . .	. . .	. . .	. . .	. . .	. . .	. . .	. . .	. . .	. . .	. . .	. . .	. . .	. . .	. . .	. . .	. . .	1074
LL37_10	. . .	. . .	. . .	. . .	. . .	. . .	. . .	. . .	. . .	. . .	. . .	. . .	. . .	. . .	. . .	. . .	. . .	. . .	. . .	. . .	. . .	. . .	. . .	. . .	. . .	. . .	. . .	. . .	. . .	. . .	1080
LL37_11	. . .	. . .	. . .	. . .	. . .	. . .	. . .	. . .	. . .	. . .	. . .	. . .	. . .	. . .	. . .	. . .	. . .	. . .	. . .	. . .	. . .	. . .	. . .	. . .	. . .	. . .	. . .	. . .	. . .	. . .	1074
LL37_12	. . .	. . .	. . .	. . .	. . .	. . .	. . .	. . .	. . .	. . .	. . .	. . .	. . .	. . .	. . .	. . .	. . .	. . .	. . .	. . .	. . .	. . .	. . .	. . .	. . .	. . .	. . .	. . .	. . .	. . .	1080
LL37_13	. . .	. . .	. . .	. . .	. . .	. . .	. . .	. . .	. . .	. . .	. . .	. . .	. . .	. . .	. . .	. . .	. . .	. . .	. . .	. . .	. . .	. . .	. . .	. . .	. . .	. . .	. . .	. . .	. . .	. . .	1080
LL37_14	. . .	. . .	. . .	. . .	. . .	. . .	. . .	. . .	. . .	. . .	. . .	. . .	. . .	. . .	. . .	. . .	. . .	. . .	. . .	. . .	. . .	. . .	. . .	. . .	. . .	. . .	. . .	. . .	. . .	. . .	1080
LL37_15	. . .	. . .	. . .	. . .	. . .	. . .	. . .	. . .	. . .	. . .	. . .	. . .	. . .	. . .	. . .	. . .	. . .	. . .	. . .	. . .	. . .	. . .	. . .	. . .	. . .	. . .	. . .	. . .	. . .	. . .	1080
LL37_16	. . .	. . .	. . .	. . .	. . .	. . .	. . .	. . .	. . .	. . .	. . .	. . .	. . .	. . .	. . .	. . .	. . .	. . .	. . .	. . .	. . .	. . .	. . .	. . .	. . .	. . .	A . .	. . .	. . .	. . .	1080
LL37_17	. . .	. . .	. . .	. . .	. . .	. . .	. . .	. . .	. . .	. . .	. . .	. . .	. . .	. . .	. . .	. . .	. . .	. . .	. . .	. . .	. . .	. . .	. . .	. . .	. . .	. . .	. . .	. . .	. . .	. . .	1080
LL37_18	. . .	. . .	. . .	. . .	. . .	. . .	. . .	. . .	. . .	. . .	. . .	. . .	. . .	. . .	. . .	. . .	. . .	. . .	. . .	. . .	. . .	. . .	. . .	. . .	. . .	. . .	. . .	. . .	. . .	. . .	1074
LL37_19	. . .	. . .	. . .	. . .	. . .	. . .	. . .	. . .	. . .	. . .	. . .	. . .	. . .	. . .	. . .	. . .	. . .	. . .	. . .	. . .	. . .	. . .	. . .	. . .	. . .	. . .	. . .	. . .	. . .	. . .	1080
LL37_20	. . .	. . .	. . .	. . .	. . .	. . .	. . .	. . .	. . .	. . .	. . .	. . .	. . .	. . .	. . .	. . .	. . .	. . .	. . .	. . .	. . .	. . .	. . .	. . .	. . .	. . .	. . .	. . .	. . .	. . .	1080
LL37Iron_1	. . .	. . .	. . .	. . .	. . .	. . .	. . .	. . .	. . .	. . .	. . .	. . .	. . .	. . .	. . .	. . .	. . .	. . .	. . .	. . .	. . .	. . .	. . .	. . .	. . .	. . .	. . .	. . .	. . .	. . .	1080
LL37Iron_2	. . .	. . .	. . .	. . .	. . .	. . .	. . .	. . .	. . .	. . .	. . .	. . .	. . .	. . .	. . .	. . .	. . .	. . .	. . .	. . .	. . .	. . .	. . .	. . .	. . .	. . .	. . .	. . .	. . .	. . .	1080
LL37Iron_3	. . .	. . .	. . .	. . .	. . .	. . .	. . .	. . .	. . .	. . .	. . .	. . .	. . .	. . .	. . .	. . .	. . .	. . .	. . .	. . .	. . .	. . .	. . .	. . .	. . .	. . .	. . .	. . .	. . .	. . .	1080
LL37Iron_4	. . .	. . .	. . .	. . .	. . .	. . .	. . .	. . .	. . .	. . .	. . .	. . .	. . .	. . .	. . .	. . .	. . .	. . .	. . .	. . .	. . .	. . .	. . .	. . .	. . .	. . .	. . .	. . .	. . .	. . .	1080
LL37Iron_5	. . .	. . .	. . .	. . .	. . .	. . .	. . .	. . .	. . .	. . .	. . .	. . .	. . .	. . .	. . .	. . .	. . .	. . .	. . .	. . .	. . .	. . .	. . .	. . .	. . .	. . .	. . .	. . .	. . .	. . .	1080
LL37Iron_6	. . .	. . .	. . .	. . .	. . .	. . .	. . .	. . .	. . .	. . .	. . .	. . .	. . .	. . .	. . .	. . .	. . .	. . .	. . .	. . .	. . .	. . .	. . .	. . .	. . .	. . .	. . .	. . .	. . .	. . .	1080
LL37Iron_7	. . .	. . .	. . .	. . .	. . .	. . .	. . .	. . .	. . .	. . .	. . .	. . .	. . .	. . .	. . .	. . .	. . .	. . .	. . .	. . .	. . .	. . .	. . .	. . .	. . .	. . .	. . .	. . .	. . .	. . .	1080
LL37Iron_8	. . .	. . .	. . .	. . .	. . .	. . .	. . .	. . .	. . .	. . .	. . .	. . .	. . .	. . .	. . .	. . .	. . .	. . .	. . .	. . .	. . .	. . .	. . .	. . .	. . .	. . .	. . .	. . .	. . .	. . .	1080
LL37Iron_9	. . .	. . .	. . .	. . .	. . .	. . .	. . .	. . .	. . .	. . .	. . .	. . .	. . .	. . .	. . .	. . .	. . .	. . .	. . .	. . .	. . .	. . .	. . .	. . .	. . .	. . .	. . .	. . .	. . .	. . .	1080
LL37Iron_10	. . .	. . .	. . .	. . .	. . .	. . .	. . .	. . .	. . .	. . .	. . .	. . .	. . .	. . .	. . .	. . .	. . .	. . .	. . .	. . .	. . .	. . .	. . .	. . .	. . .	. . .	. . .	. . .	. . .	. . .	1080
LL37Iron_11	. . .	. . .	. . .	. . .	. . .	. . .	. . .	. . .	. . .	. . .	. . .	. . .	. . .	. . .	. . .	. . .	. . .	. . .	. . .	. . .	. . .	. . .	. . .	. . .	. . .	. . .	. . .	. . .	. . .	. . .	1080
LL37Iron_12	. . .	. . .	. . .	. . .	. . .	. . .	. . .	. . .	. . .	. . .	. . .	. . .	. . .	. . .	. . .	. . .	. . .	. . .	. . .	. . .	. . .	. . .	. . .	. . .	. . .	. . .	. . .	. . .	. . .	. . .	1080
LL37Iron_13	. . .	. . .	. . .	. . .	. . .	. . .	. . .	. . .	. . .	. . .	. . .	. . .	. . .	. . .	. . .	. . .	. . .	. . .	. . .	. . .	. . .	. . .	. . .	. . .	. . .	. . .	. . .	. . .	. . .	. . .	1080
LL37Iron_14	. . .	. . .	. . .	. . .	. . .	. . .	. . .	. . .	. . .	. . .	. . .	. . .	. . .	. . .	. . .	. . .	. . .	. . .	. . .	. . .	. . .	. . .	. . .	. . .	. . .	. . .	. . .	. . .	. . .	. . .	1080
LL37Iron_15	. . .	. . .	. . .	. . .	. . .	. . .	. . .	. . .	. . .	. . .	. . .	. . .	. . .	. . .	. . .	. . .	. . .	. . .	. . .	. . .	. . .	. . .	. . .	. . .	. . .	. . .	. . .	. . .	. . .	. . .	1080
LL37Iron_16	. . .	. . .	. . .	. . .	. . .	. . .	. . .	. . .	. . .	. . .	. . .	. . .	. . .	. . .	. . .	. . .	. . .	. . .	. . .	. . .	. . .	. . .	. . .	. . .	. . .	. . .	. . .	. . .	. . .	. . .	1080
LL37Iron_17	. . .	. . .	. . .	. . .	. . .	. . .	. . .	. . .	. . .	. . .	. . .	. . .	. . .	. . .	. . .	. . .	. . .	. . .	. . .	. . .	. . .	. . .	. . .	. . .	. . .	. . .	. . .	. . .	. . .	. . .	1080

LL37Iron_18 1079
LL37Iron_19 1080
LL37Iron_20 1080

						1.100							1.120						1.140							1.160	
WT_PA14_orf	AAG	AAA	GCG	GCC	GGC	ACC	GCC	GCC	GGC	TTC	ACC	GGT	CTG	TTC	GGC	TAC	CTC	GGC	GGC	TCG	GTG	GCA	GCC	AAC	GCC	CTG	GTC	1161
iron_1	. . .	. . .	. . .	. . .	. . .	. . .	. . .	. . .	. . .	. . .	. . .	. . .	. . .	. . .	. . .	. . .	. . .	. . .	. . .	. . .	. . .	. . .	. . .	. . .	. . .	. . .	. . .	1161
iron_2	. . .	. . .	. . .	. . .	. . .	. . .	. . .	. . .	. . .	. . .	. . .	. . .	. . .	. . .	. . .	. . .	. . .	. . .	. . .	. . .	. . .	. . .	. . .	. . .	. . .	. . .	. . .	1155
iron_3	. . .	. . .	. . .	. . .	. . .	. . .	. . .	. . .	. . .	. . .	. . .	. . .	. . .	. . .	. . .	. . .	. . .	. . .	. . .	. . .	. . .	. . .	. . .	. . .	. . .	. . .	. . .	1161
iron_4	. . .	. . .	. . .	. . .	. . .	. . .	. . .	. . .	. . .	. . .	. . .	. . .	. . .	. . .	. . .	. . .	. . .	. . .	. . .	. . .	. . .	. . .	. . .	. . .	. . .	. . .	. . .	1161
iron_5	. . .	. . .	. . .	. . .	. . .	. . .	. . .	. . .	. . .	. . .	. . .	. . .	. . .	. . .	. . .	. . .	. . .	. . .	. . .	. . .	. . .	. . .	. . .	. . .	. . .	. . .	. . .	1161
iron_6	. . .	. . .	. . .	. . .	. . .	. . .	. . .	. . .	. . .	. . .	. . .	. . .	. . .	. . .	. . .	. . .	. . .	. . .	. . .	. . .	. . .	. . .	. . .	. . .	. . .	. . .	. . .	1161
iron_7	. . .	. . .	. . .	. . .	. . .	. . .	. . .	. . .	. . .	. . .	. . .	. . .	. . .	. . .	. . .	. . .	. . .	. . .	. . .	. . .	. . .	. . .	. . .	. . .	. . .	. . .	. . .	1161
iron_8	. . .	. . .	. . .	. . .	. . .	. . .	. . .	. . .	. . .	. . .	. . .	. . .	. . .	. . .	. . .	. . .	. . .	. . .	. . .	. . .	. . .	. . .	. . .	. . .	. . .	. . .	. . .	1161
iron_9	. . .	. . .	. . .	. . .	. . .	. . .	. . .	. . .	. . .	. . .	. . .	. . .	. . .	. . .	. . .	. . .	. . .	. . .	. . .	. . .	. . .	. . .	. . .	. . .	. . .	. . .	. . .	1161
iron_10	. . .	. . .	. . .	. . .	. . .	. . .	. . .	. . .	. . .	. . .	. . .	. . .	. . .	. . .	. . .	. . .	. . .	. . .	. . .	. . .	. . .	. . .	. . .	. . .	. . .	. . .	. . .	1155
iron_11	. . .	. . .	. . .	. . .	. . .	. . .	. . .	. . .	. . .	. . .	. . .	. . .	. . .	. . .	. . .	. . .	. . .	. . .	. . .	. . .	. . .	. . .	. . .	. . .	. . .	. . .	. . .	1155
iron_12	. . .	. . .	. . .	. . .	. . .	. . .	. . .	. . .	. . .	. . .	. . .	. . .	. . .	. . .	. . .	. . .	. . .	. . .	. . .	. . .	. . .	. . .	. . .	. . .	. . .	. . .	. . .	1161
iron_13	. . .	. . .	. . .	. . .	. . .	. . .	. . .	. . .	. . .	. . .	. . .	. . .	. . .	. . .	. . .	. . .	. . .	. . .	. . .	. . .	. . .	. . .	. . .	. . .	. . .	. . .	. . .	1155
iron_14	. . .	. . .	. . .	. . .	. . .	. . .	. . .	. . .	. . .	. . .	. . .	. . .	. . .	. . .	. . .	. . .	. . .	. . .	. . .	. . .	. . .	. . .	. . .	. . .	. . .	. . .	. . .	1155
iron_15	. . .	. . .	. . .	. . .	. . .	. . .	. . .	. . .	. . .	. . .	. . .	. . .	. . .	. . .	. . .	. . .	. . .	. . .	. . .	. . .	. . .	. . .	. . .	. . .	. . .	. . .	. . .	1155
iron_16	. . .	. . .	. . .	. . .	. . .	. . .	. . .	. . .	. . .	. . .	. . .	. . .	. . .	. . .	. . .	. . .	. . .	. . .	. . .	. . .	. . .	. . .	. . .	. . .	. . .	. . .	. . .	1155
iron_17	. . .	. . .	. . .	. . .	. . .	. . .	. . .	. . .	. . .	. . .	. . .	. . .	. . .	. . .	. . .	. . .	. . .	. . .	. . .	. . .	. . .	. . .	. . .	. . .	. . .	. . .	. . .	1155
iron_18	. . .	. . .	. . .	. . .	. . .	. . .	. . .	. . .	. . .	. . .	. . .	. . .	. . .	. . .	. . .	. . .	. . .	. . .	. . .	. . .	. . .	. . .	. . .	. . .	. . .	. . .	. . .	1161
iron_19	. . .	. . .	. . .	. . .	. . .	. . .	. . .	. . .	. . .	. . .	. . .	. . .	. . .	. . .	. . .	. . .	. . .	. . .	. . .	. . .	. . .	. . .	. . .	. . .	. . .	. . .	. . .	1161
iron_20	. . .	. . .	. . .	. . .	. . .	. . .	. . .	. . .	. . .	. . .	. . .	. . .	. . .	. . .	. . .	. . .	. . .	. . .	. . .	. . .	. . .	. . .	. . .	. . .	. . .	. . .	. . .	1161
LL37_1	. . .	. . .	. . .	. . .	. . .	. . .	. . .	. . .	. . .	. . .	. . .	. . .	. . .	. . .	. . .	. . .	. . .	. . .	. . .	. . .	. . .	. . .	. . .	. . .	. . .	. . .	. . .	1161
LL37_2	. . .	. . .	. . .	. . .	. . .	. . .	. . .	. . .	. . .	. . .	. . .	. . .	. . .	. . .	. . .	. . .	. . .	. . .	. . .	. . .	. . .	. . .	. . .	. . .	. . .	. . .	. . .	1155
LL37_3	. . .	. . .	. . .	. . .	. . .	. . .	. . .	. . .	. . .	. . .	. . .	. . .	. . .	. . .	. . .	. . .	. . .	. . .	. . .	. . .	. . .	. . .	. . .	. . .	. . .	. . .	. . .	1161
LL37_4	. . .	. . .	. . .	. . .	. . .	. . .	. . .	. . .	. . .	. . .	. . .	. . .	. . .	. . .	. . .	. . .	. . .	. . .	. . .	. . .	. . .	. . .	. . .	. . .	. . .	. . .	. . .	1161
LL37_5	. . .	. . .	. . .	. . .	. . .	. . .	. . .	. . .	. . .	. . .	. . .	. . .	. . .	. . .	. . .	. . .	. . .	. . .	. . .	. . .	. . .	. . .	. . .	. . .	. . .	. . .	. . .	1155
LL37_6	. . .	. . .	. . .	. . .	. . .	. . .	. . .	. . .	. . .	. . .	. . .	. . .	. . .	. . .	. . .	. . .	. . .	. . .	. . .	. . .	. . .	. . .	. . .	. . .	. . .	. . .	. . .	1161
LL37_7	. . .	. . .	. . .	. . .	. . .	. . .	. . .	. . .	. . .	. . .	. . .	. . .	. . .	. . .	. . .	. . .	. . .	. . .	. . .	. . .	. . .	. . .	. . .	. . .	. . .	. . .	. . .	1155
LL37_8	. . .	. . .	. . .	. . .	. . .	. . .	. . .	. . .	. . .	. . .	. . .	. . .	. . .	. . .	. . .	. . .	. . .	. . .	. . .	. . .	. . .	. . .	. . .	. . .	. . .	. . .	. . .	1161
LL37_9	. . .	. . .	. . .	. . .	. . .	. . .	. . .	. . .	. . .	. . .	. . .	. . .	. . .	. . .	. . .	. . .	. . .	. . .	. . .	. . .	. . .	. . .	. . .	. . .	. . .	. . .	. . .	1155
LL37_10	. . .	. . .	. . .	. . .	. . .	. . .	. . .	. . .	. . .	. . .	. . .	. . .	. . .	. . .	. . .	. . .	. . .	. . .	. . .	. . .	. . .	. . .	. . .	. . .	. . .	. . .	. . .	1161
LL37_11	. . .	. . .	. . .	. . .	. . .	. . .	. . .	. . .	. . .	. . .	. . .	. . .	. . .	. . .	. . .	. . .	. . .	. . .	. . .	. . .	. . .	. . .	. . .	. . .	. . .	. . .	. . .	1155
LL37_12	. . .	. . .	. . .	. . .	. . .	. . .	. . .	. . .	. . .	. . .	. . .	. . .	. . .	. . .	. . .	. . .	. . .	. . .	. . .	. . .	. . .	. . .	. . .	. . .	. . .	. . .	. . .	1161
LL37_13	. . .	. . .	. . .	. . .	. . .	. . .	. . .	. . .	. . .	. . .	. . .	. . .	. . .	. . .	. . .	. . .	. . .	. . .	. . .	. . .	. . .	. . .	. . .	. . .	. . .	. . .	. . .	1161
LL37_14	. . .	. . .	. . .	. . .	. . .	. . .	. . .	. . .	. . .	. . .	. . .	. . .	. . .	. . .	. . .	. . .	. . .	. . .	. . .	. . .	. . .	. . .	. . .	. . .	. . .	. . .	. . .	1161
LL37_15	. . .	. . .	. . .	. . .	. . .	. . .	. . .	. . .	. . .	. . .	. . .	. . .	. . .	. . .	. . .	. . .	. . .	. . .	. . .	. . .	. . .	. . .	. . .	. . .	. . .	. . .	. . .	1161
LL37_16	. . .	. . .	. . .	. . .	. . .	. . .	. . .	. . .	. . .	. . .	. . .	. . .	. . .	. . .	. . .	. . .	. . .	. . .	. . .	. . .	. . .	. . .	. . .	. . .	. . .	. . .	. . .	1161
LL37_17	. . .	. . .	. . .	. . .	. . .	. . .	. . .	. . .	. . .	. . .	. . .	. . .	. . .	. . .	. . .	. . .	. . .	. . .	. . .	. . .	. . .	. . .	. . .	. . .	. . .	. . .	. . .	1161
LL37_18	. . .	. . .	. . .	. . .	. . .	. . .	. . .	. . .	. . .	. . .	. . .	. . .	. . .	. . .	. . .	. . .	. . .	. . .	. . .	. . .	. . .	. . .	. . .	. . .	. . .	. . .	. . .	1155
LL37_19	. . .	. . .	. . .	. . .	. . .	. . .	. . .	. . .	. . .	. . .	. . .	. . .	. . .	. . .	. . .	. . .	. . .	. . .	. . .	. . .	. . .	. . .	. . .	. . .	. . .	. . .	. . .	1161
LL37_20	. . .	. . .	. . .	. . .	. . .	. . .	. . .	. . .	. . .	. . .	. . .	. . .	. . .	. . .	. . .	. . .	. . .	. . .	. . .	. . .	. . .	. . .	. . .	. . .	. . .	. . .	. . .	1161
LL37Iron_1	. . .	. . .	. . .	. . .	. . .	. . .	. . .	. . .	. . .	. . .	. . .	. . .	. . .	. . .	. . .	. . .	. . .	. . .	. . .	. . .	. . .	. . .	. . .	. . .	. . .	. . .	. . .	1161
LL37Iron_2	. . .	. . .	. . .	. . .	. . .	. . .	. . .	. . .	. . .	. . .	. . .	. . .	. . .	. . .	. . .	. . .	. . .	. . .	. . .	. . .	. . .	. . .	. . .	. . .	. . .	. . .	. . .	1161
LL37Iron_3	. . .	. . .	. . .	. . .	. . .	. . .	. . .	. . .	. . .	. . .	. . .	. . .	. . .	. . .	. . .	. . .	. . .	. . .	. . .	. . .	. . .	. . .	. . .	. . .	. . .	. . .	. . .	1161
LL37Iron_4	. . .	. . .	. . .	. . .	. . .	. . .	. . .	. . .	. . .	. . .	. . .	. . .	. . .	. . .	. . .	. . .	. . .	. . .	. . .	. . .	. . .	. . .	. . .	. . .	. . .	. . .	. . .	1161
LL37Iron_5	. . .	. . .	. . .	. . .	. . .	. . .	. . .	. . .	. . .	. . .	. . .	. . .	. . .	. . .	. . .	. . .	. . .	. . .	. . .	. . .	. . .	. . .	. . .	. . .	. . .	. . .	. . .	1161
LL37Iron_6	. . .	. . .	. . .	. . .	. . .	. . .	. . .	. . .	. . .	. . .	. . .	. . .	. . .	. . .	. . .	. . .	. . .	A . .	. . .	. . .	. . .	. . .	. . .	. . .	. . .	. . .	. . .	1161
LL37Iron_7	. . .	. . .	. . .	. . .	. . .	. . .	. . .	. . .	. . .	. . .	. . .	. . .	. . .	. . .	. . .	. . .	. . .	. . .	. . .	. . .	. . .	. . .	. . .	. . .	. . .	. . .	. . .	1161
LL37Iron_8	. . .	. . .	. . .	. . .	. . .	. . .	. . .	. . .	. . .	. . .	. . .	. . .	. . .	. . .	. . .	. . .	. . .	. . .	. . .	. . .	. . .	. . .	. . .	. . .	. . .	. . .	. . .	1161
LL37Iron_9	. . .	. . .	. . .	. . .	. . .	. . .	. . .	. . .	. . .	. . .	. . .	. . .	. . .	. . .	. . .	. . .	. . .	. . .	. . .	. . .	. . .	. . .	. . .	. . .	. . .	. . .	. . .	1161
LL37Iron_10	. . .	. . .	. . .	. . .	. . .	. . .	. . .	. . .	. . .	. . .	. . .	. . .	. . .	. . .	. . .	. . .	. . .	. A .	. . .	. . .	. . .	. . .	. . .	. . .	. . .	. . .	. . .	1161
LL37Iron_11	. . .	. . .	. . .	. . .	. . .	. . .	. . .	. . .	. . .	. . .	. . .	. . .	. . .	. . .	. . .	. . .	. . .	. . .	. . .	. . .	. . .	. . .	. . .	. . .	. . .	. . .	. . .	1161
LL37Iron_12	. . .	. . .	. . .	. . .	. . .	. . .	. . .	. . .	. . .	. . .	. . .	. . .	. . .	. . .	. . .	. . .	. . .	. . .	. . .	. . .	. . .	. . .	. . .	. . .	. . .	. . .	. . .	1161
LL37Iron_13	. . .	. . .	. . .	. . .	. . .	. . .	. . .	. . .	. . .	. . .	. . .	. . .	. . .	. . .	. . .	. . .	. . .	. . .	. . .	. . .	. . .	. . .	. . .	. . .	. . .	. . .	. . .	1161
LL37Iron_14	. . .	. . .	. . .	. . .	. . .	. . .	. . .	. . .	. . .	. . .	. . .	. . .	. . .	. . .	. . .	. . .	. . .	. . .	. . .	. . .	. . .	. . .	. . .	. . .	. . .	. . .	. . .	1161
LL37Iron_15	. . .	. . .	. . .	. . .	. . .	. . .	. . .	. . .	. . .	. . .	. . .	. . .	. . .	. . .	. . .	. . .	. . .	. . .	. . .	. . .	. . .	. . .	. . .	. . .	. . .	. . .	. . .	1161
LL37Iron_16	. . .	. . .	. . .	. . .	. . .	. . .	. . .	. . .	. . .	. . .	. . .	. . .	. . .	. . .	. . .	. . .	. . .	. . .	. . .	. . .	. . .	. . .	. . .	. . .	. . .	. . .	. . .	1161
LL37Iron_17	. . .	. . .	. . .	. . .	. . .	. . .	. . .	. . .	. . .	. . .	. . .	. . .	. . .	. . .	. . .	. . .	. . .	. . .	. . .	. . .	. . .	. . .	. . .	. . .	. . .	. . .	. . .	1161

LL37Iron_18 1160
LL37Iron_19 . . . . A 1161
LL37Iron_20 1161
