# Supplementary material for: Cationic Peptides Facilitate Iron-induced Mutagenesis in Bacteria
Source: PLoS Genet. 2015 Oct 2;11(10):e1005546. doi: 10.1371/journal.pgen.1005546 (PMC4592263; doi:10.1371/journal.pgen.1005546)
Supplement: S1 Table — (PDF) [file pgen.1005546.s008.pdf]

Table S1. Mutations in *glpT* of Fos-R *P. aeruginosa* PA14 clones treated with iron, LL-37 and LL-37+Fe^{2+}

Treatment	Mutant number	Mutation position[1]	Amino acid change[2]	Protein stability ddG Value[3]	Protein domain[4]
Iron	1	G230 to A transition	G77 to D	-1.09 Large Decrease of Stability	TMhelix
	2	A220TCGCC225, deletion 6 bp	deletion of I74- A75	NA	TMhelix
	3	A846 to T transversion	E282 to V	0.08 Increase of Stability	outside
	4	G185 to A transition	R62 to H	-1.33 Large Decrease of Stability	outside
	5	A846 to T transversion	E282 to V	0.08 Increase of Stability	outside
	6	G250 to T transversion	G84 to C	-1.38Large Decrease of Stability	TMhelix
	7	G409 to A	G137 to S	-1.01 Large Decrease of Stability	TMhelix
	8	T908 to C transition	I303 to T	-2.05 Large Decrease of Stability	TMhelix
	9	T823 to A transversion	W275 to R	-1.09 Large Decrease of Stability	outside
	10	A220TCGCC225, deletion 6 bp	deletion of I74- A75	NA	TMhelix
	11	A220TCGCC225, deletion 6 bp	deletion of I74- A75	NA	TMhelix
	12	T149 to C transition	L50 to P	-1.91 Large Decrease of Stability	outside
	13	A220TCGCC225, deletion 6 bp	deletion of I74- A75	NA	TMhelix
	14	A220TCGCC225, deletion 6 bp	deletion of I74- A75	NA	TMhelix
	15	A220TCGCC225, deletion 6 bp	deletion of I74- A75	NA	TMhelix
	16	A220TCGCC225, deletion 6 bp	deletion of I74- A75	NA	TMhelix
	17	A220TCGCC225, deletion 6 bp	deletion of I74- A75	NA	TMhelix
	18	G409 to T	G137 to C	-0.84 Large Decrease of Stability	TMhelix
	19	T524 to C transition	L175 to P	-1.06 Large Decrease of Stability	TMhelix
	20	G647 to A transition	G216 to D	-1.03 Large Decrease of Stability	inside
LL-37	1	A496 to G transition	N166 to D	-0.08 Decrease of Stability	TMhelix
	2	A220TCGCC225, deletion 6 bp	deletion of I74- A75	NA	TMhelix
	3	A1006 to C transversion	T336 to P	-0.70 Large Decrease of Stability	TMhelix
	4	A401 to T transversion	Q134 to L	-0.10 Decrease of Stability	TMhelix
	5	A220TCGCC225, deletion 6 bp	deletion of I74- A75	NA	TMhelix
	6	T524 to C transition	L175 to P	-1.06 Large Decrease of Stability	TMhelix
	7	A220TCGCC225, deletion 6 bp	deletion of I74- A75	NA	TMhelix
	8	C894 to A transversion, stop codon	Y298 to stop , truncation	NA	NA
	9	A220TCGCC225, deletion 6 bp	deletion of I74- A75	NA	TMhelix

	10	G98 to A transition	G33 to D	-0.95,Large Decrease of Stability	TMhelix
	11	A220TCGCC225, deletion 6 bp	deletion of I74- A75	NA	TMhelix
	12	G994 to C transversion	V332 to L	-1.30 Large Decrease of Stability	TMhelix
	13	G457 to A transition	E153 to K	-0.70 Large Decrease of Stability	inside
	14	G409 to T	G137 to C	-0.84 Large Decrease of Stability	TMhelix
	15	A430 to C transversion	T144 to P	-0.39 Decrease of Stability	inside
	16	G1120 to A transition	E374 to K	-0.49 Decrease of Stability	Inside
	17	G824 to A transition, stop codon	W275 to stop , truncation	NA	outside
	18	A220TCGCC225, deletion 6 bp	deletion of I74- A75	NA	TMhelix
	19	G913 to A transition	G305 to S	-1.10 Large Decrease of Stability	TMhelix
	20	G229 to A transition	G77 to S	-1.23 Large Decrease of Stability	TMhelix
LL-37+Fe^{2+}	1	G403 to A transition	G135 to S	-1.05 Large Decrease of Stability	TMhelix
	2	C277 to T transition	R93 to W	-0.57 Large Decrease of Stability	inside
	2	G403 to T transversion	G135 to C	-0.90 Large Decrease of Stability	TMhelix
	3	C822 to G transversion	D274 to E	-0.08 Decrease of Stability	outside
	4	C277 to T transition	R93 to W	-0.57 Large Decrease of Stability	inside
	5	C277 to T transition	R93 to W	-0.57 Large Decrease of Stability	inside
	6	G1183 to A transition	G395 to S	-1.20 Large Decrease of Stability	TMhelix
	7	C277 to T transition	R93 to W	-0.57 Large Decrease of Stability	inside
	8	C277 to T transition	R93 to W	-0.57 Large Decrease of Stability	inside
	9	C277 to T transition	R93 to W	-0.57 Large Decrease of Stability	inside
	10	G1184 to A transition	G395 to D	-0.85 Large Decrease of Stability	TMhelix
	11	C491 to T transition	A164 to V	0.01 Increase of Stability	TMhelix
	12	C491 to T transition	A164 to V	0.01 Increase of Stability	TMhelix
	13	C822 to G transversion	D274 to E	-0.08 Decrease of Stability	outside
	14	C277 to T transition	R93 to W	-0.57 Large Decrease of Stability	inside
	15	C277 to T transition	R93 to W	-0.57 Large Decrease of Stability	inside
	16	C277 to T transition	R93 to W	-0.57 Large Decrease of Stability	inside
	17	C277 to T transition	R93 to W	-0.57 Large Decrease of Stability	inside
	18	T299, deletion 1bp, frameshift	frameshift	NA	NA

19	G1183 to A transition	G395	to S	-1.20 Large Decrease of Stability	TMhelix
20	G403 to T transition	G135	to C	-0.90 Large Decrease of Stability	TMhelix

[1] relative to the A in the start ATG codon of the 1347bp-long *glpT* ORF

[2] relative to the first amino acid of the GlpT protein

[3] ddG values are calculated using I-Mutant3.0 software for prediction of the protein stability change upon mutation, available from http://gpcr.biocomp.unibo.it/~emidio/I-Mutant3.0/I-MutantDDG_Help.html

[4] location of transmembrane helices in proteins was predicted using TMHMM Server v. 2.0, available from http://www.cbs.dtu.dk/services/TMHMM-2.0/
