# Supplementary material for: Cationic Peptides Facilitate Iron-induced Mutagenesis in Bacteria
Source: PLoS Genet. 2015 Oct 2;11(10):e1005546. doi: 10.1371/journal.pgen.1005546 (PMC4592263; doi:10.1371/journal.pgen.1005546)
Supplement: S4 Table — H0 is rejected if P<0.05. Mutation hotspots that significantly different are highlighted. (PDF) [file pgen.1005546.s011.pdf]

Table S4.

Mutation site	Number of mutants per treatment		Two-tailed *P*-value	Interpretation
	Iron	LL-37+Fe^{2+}		
G33 to D	0	0	> 0.9999	The groups are not significantly different.
L50 to P	1	0	> 0.9999	The groups are not significantly different.
R62 to H	1	0	> 0.9999	The groups are not significantly different.
deletion of 6 bp I74 - A75	8	0	0.0033	The groups are significantly different.
G77 to D	1	0	> 0.9999	The groups are not significantly different.
G84 to C	1	0	> 0.9999	The groups are not significantly different.
R93 to W	0	10	0.0004	The groups are significantly different.
Q134 to L	0	0	> 0.9999	The groups are not significantly different.
G135 to S	0	3	0.2308	The groups are not significantly different.
G137 to S	2	0	0.4872	The groups are not significantly different.
T299 frameshift	0	1	> 0.9999	The groups are not significantly different.
T144 to P	0	0	> 0.9999	The groups are not significantly different.
E153 to K	0	0	> 0.9999	The groups are not significantly different.
N166 to D	0	0	> 0.9999	The groups are not significantly different.
A164 to V	0	2	0.4872	The groups are not significantly different.
L175 to P	1	0	> 0.9999	The groups are not significantly different.
G216 to D	1	0	> 0.9999	The groups are not significantly different.
D274 to E	0	2	0.4872	The groups are not significantly different.
W275 to R	1	0	> 0.9999	The groups are not significantly different.
E282 to V	2	0	> 0.9999	The groups are not significantly different.
Y298 to stop, truncation	0	0	> 0.9999	The groups are not significantly different.
I303 to T	1	0	> 0.9999	The groups are not significantly different.
G305 to S	0	0	> 0.9999	The groups are not significantly different.
V332 to L	0	0	> 0.9999	The groups are not significantly different.
T336 to P	0	0	> 0.9999	The groups are not significantly different.
E374 to K	0	0	> 0.9999	The groups are not significantly different.
G395 to S	0	3	0.2308	The groups are not significantly different.
